# Supplementary material for: The halogen bond with isocyano carbon reduces isocyanide odor
Source: Nat Commun. 2020 Jun 10;11:2921. doi: 10.1038/s41467-020-16748-x (PMC7286913; doi:10.1038/s41467-020-16748-x)
Supplement: Supplementary file 1 — Supplementary Information [file 41467_2020_16748_MOESM1_ESM.pdf]

Supplementary Information

The Halogen Bond with Isocyano Carbon Reduces Isocyanide Odor

A.S. Mikherdov *et al.*

(IAd)•IPFB adduct. The CSD search indicates that the shortest contact between the I and C centers is the adduct of IPFB with the stable N-heterocyclic carbene IAd[2] of the push-push type[3] (dXB = 2.754(3) Å; 75% of ∑BvdW; CCDC: YABJAM; **Supplementary Figure 1**); IAd exhibits very strong donor properties that determine the strong I•••C linkage. Based on geometrical parameters such interaction could be considered as XB.[4]

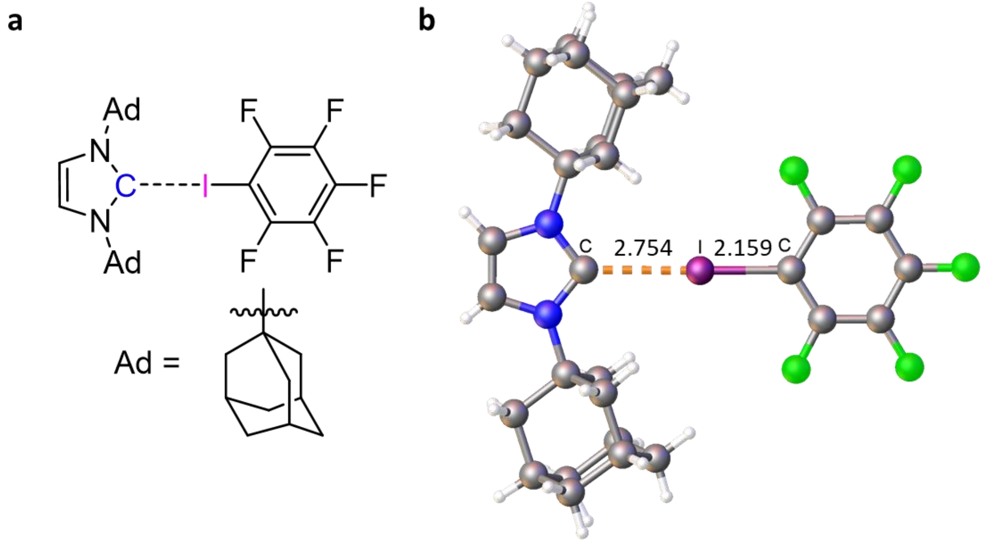

Supplementary Figure 1 Structure of (IAd)•IPFB adduct: **a** schematic view and **b** XRD structure (CCDC: YABJAM)

To reveal the nature of the I•••C contact in the (IAd)•IPFB adduct and quantify the energy of this interaction from theoretical viewpoint, we carried out DFT calculations and performed topological analysis of the electron density distribution within the framework of Bader's theory (QTAIM method)[5] for the experimental XRD geometry of the adduct as well as for their optimized equilibrium geometry in the gas phase. The full geometry optimization of (IAd)•IPFB adduct and single point calculations based on the experimental XRD data have been carried out at the DFT level of theory using the M06-2X functional[6] and CEP-121G basis sets[7,8] with the help of the Gaussian-09 program package.[9] Results of

QTAIM analysis are summarized in **Supplementary Table 1**. The contour line diagram of the Laplacian distribution $\nabla^2\rho(\mathbf{r})$, bond paths, and selected zero-flux surfaces, and reduced density gradient (RDG) isosurface for I•••C intermolecular noncovalent contact in the adduct are shown in **Supplementary Figure 2**.

Supplementary Table 1 Values of the density of all electrons – ρ(r), Laplacian of electron density – $\nabla^2\rho(\mathbf{r})$, energy density – H_b, potential energy density – V(r), and Lagrangian kinetic energy – G(r) (a.u.) at the bond critical point (3, –1), corresponding to XBs in XRD and optimized gas phase equilibrium structures of (IAd)•IPFB adduct, bond lengths – *d* (Å), Wiberg bond indexes (WI), as well as energies for these contacts E_{int} (kcal/mol), defined by different approaches

Contact	ρ(**r**)	$\nabla^2\rho(\mathbf{r})$	H_b	V(**r**)	G(**r**)	E_{int}^a	E_{int}^b	E_{int}^c	E_{int}^d	*d*	WI
(IAd)•IPFB (XRD)											
I1D•••C1A	0.037	0.084	-0.003	-0.024	0.021	7.5	5.7	10.2	8.8	2.754	0.25
(IAd)•IPFB (gas phase)											
I1D•••C1A	0.044	0.093	-0.006	-0.030	0.024	9.4	6.5	12.8	10.1	2.649	0.31

[a] $E_{int} = -V(\mathbf{r})/2$;[10] [b] $E_{int} = 0.429G(\mathbf{r})$;[11]
[c] $E_{int} = 0.68(-V(\mathbf{r}))$;[12] [d] $E_{int} = 0.67G(\mathbf{r})$;[12]

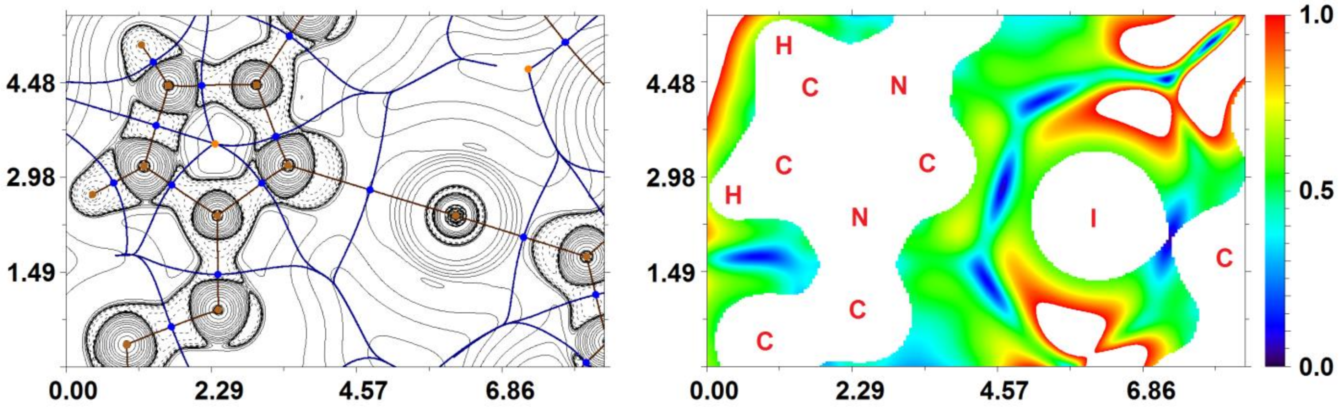

Supplementary Figure 2 Contour line diagram of the Laplacian distribution $\nabla^2\rho(\mathbf{r})$, bond paths and selected zero-flux surfaces (left) and RDG isosurface (right) referring to I•••C noncovalent interaction in the (IAd)•IPFB adduct. Bond critical points (3, –1) are shown in blue, nuclear critical points (3, –3) – in pale brown, ring critical points (3, +1) – in orange. Length units – Å, RDG isosurface values are given in a.u.

Results of QTAIM analysis reveal that the I•••C interaction in the (IAd)•IPFB adduct is much stronger than these in cases of isocyanide adducts discussed in the main text. Furthermore, this contact in (IAd)•IPFB has significant degree of covalency what follows from the relation |V(**r**)| > G(**r**) in the appropriate bond critical points (3, –1) and large values of corresponding Wiberg bond indices (WBI),[13] viz. 0.25 (for experimental XRD geometry) and 0.31 (for gas phase optimized geometry) of of normal single covalent bond. This values are closer to the WBI of the classic example of coordinative [N–I–N]+ XB in bis(pyridine)iodonium salts (WBI is 0.43)[14] than to one of the adduct with isocyanide (CNMes)•IPFB (0.1). The calculated vertical and adiabatic total energies for the model supramolecular associate (IAd)•IPFB dissociation in the gas phase are 22.3 and 17.8 kcal/mol, respectively (**Supplementary Table 2**). We also defined the direction of charge transfer (CT) in this system by NBO analysis.[15] Second order perturbation theory analysis of Fock matrix in NBO basis reveals also two direction of intermolecular CT along the I•••C interaction in the optimized equilibrium structure of (IAd)•IPFB adduct: the CT lp($C_{carbene}$) → σ*(C−I_{IPFB}) with appropriate total E(2) values 30.00 kcal/mol and transfer lp(I_{IPFB}) → σ*/π*(C=$N_{carbene}$) with appropriate total E(2) values 7.72 kcal/mol. The obtained values for the for the dissociation energies and CT are not dramatically lower than the dissociation energies for the covalent C–I bond with ca. 40–55 kcal/mol.[16] Thus, the observed I•••C interaction in (IAd)•IPFB adduct should not be considered as noncovalent due to the high energy and significant degree of covalency.

Supplementary Table 2 Vertical and adiabatic dissociation energies for gas phase optimized structures of (IAd)•IPFB adduct

Approach for energy estimation	Total electronic energies, a.u.			Dissociation energy, kcal/mol
	XB acceptor	XB donor	Adduct	
Vertical	–168.662977083	–165.995827608	–334.694382591	22.3
Adiabatic	–168.666403248	–165.999683890	–334.694382591	17.8

X-ray structures of the XB adducts with isocyanides

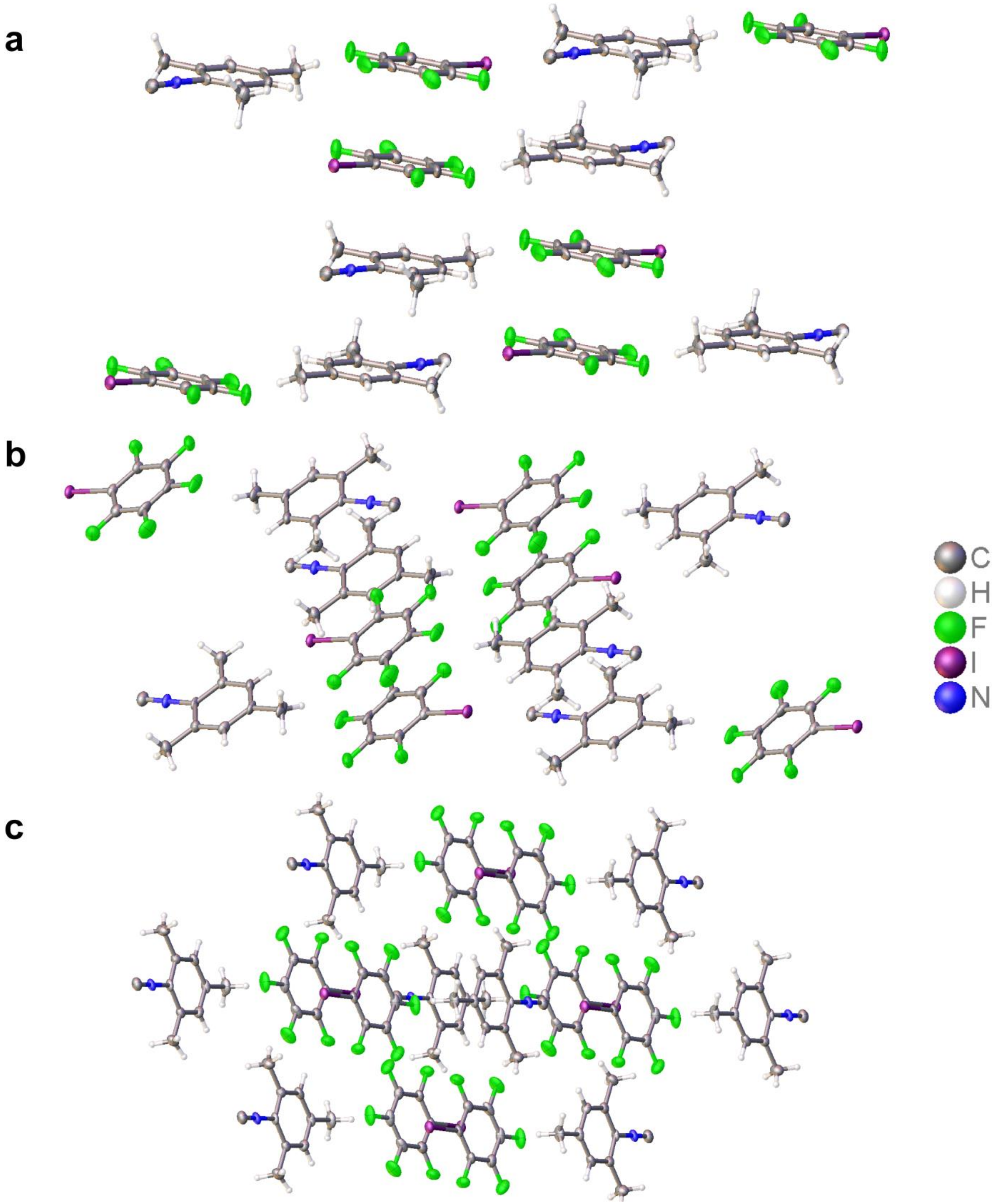

Supplementary Figure 3 Views along **a**, **b**, and **c** crystallographic axis of (CNMes)•IPFB.

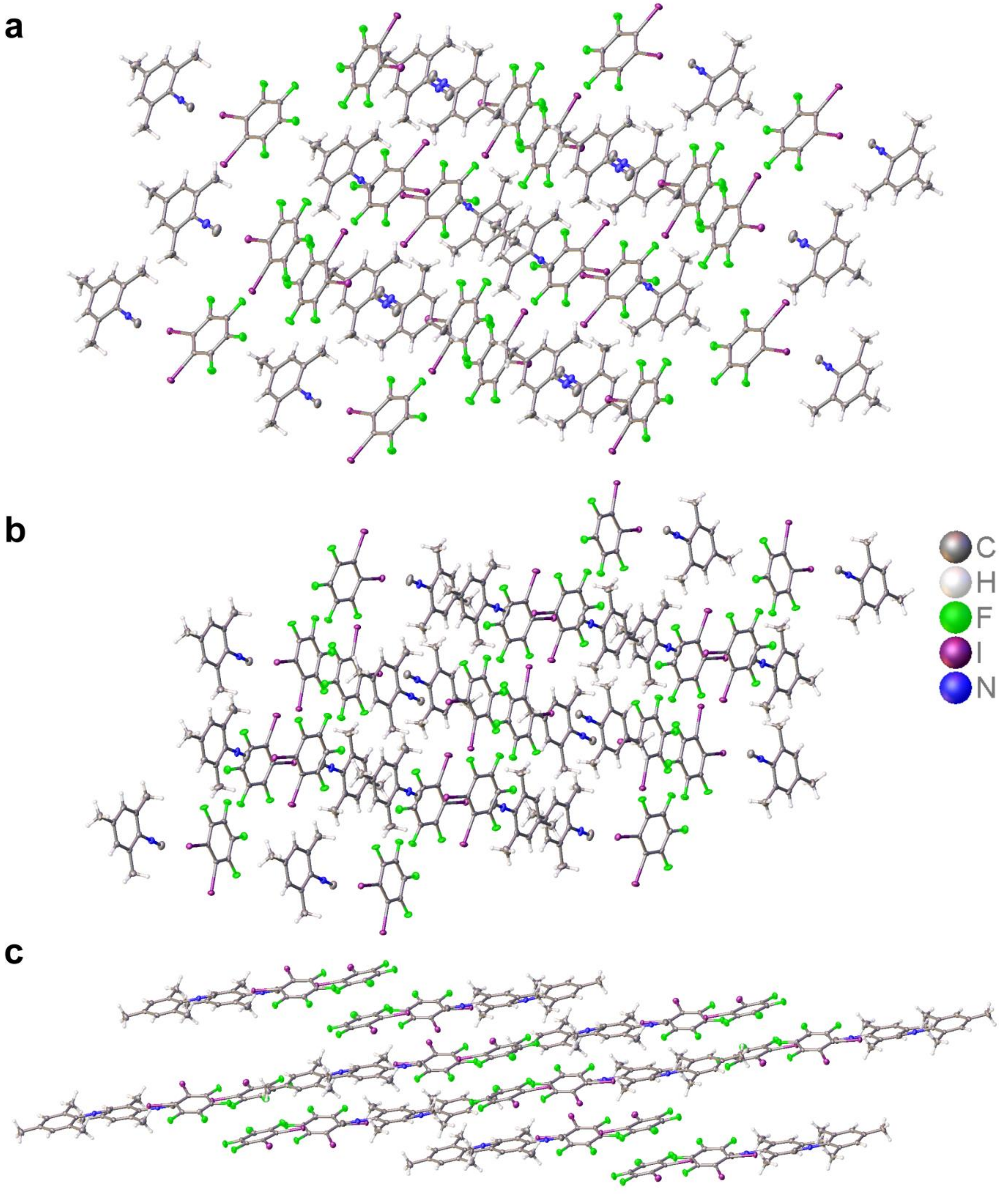

Supplementary Figure 4 Views along **a**, **b**, and **c** crystallographic axis of (CNMes)•1,2-FIB.

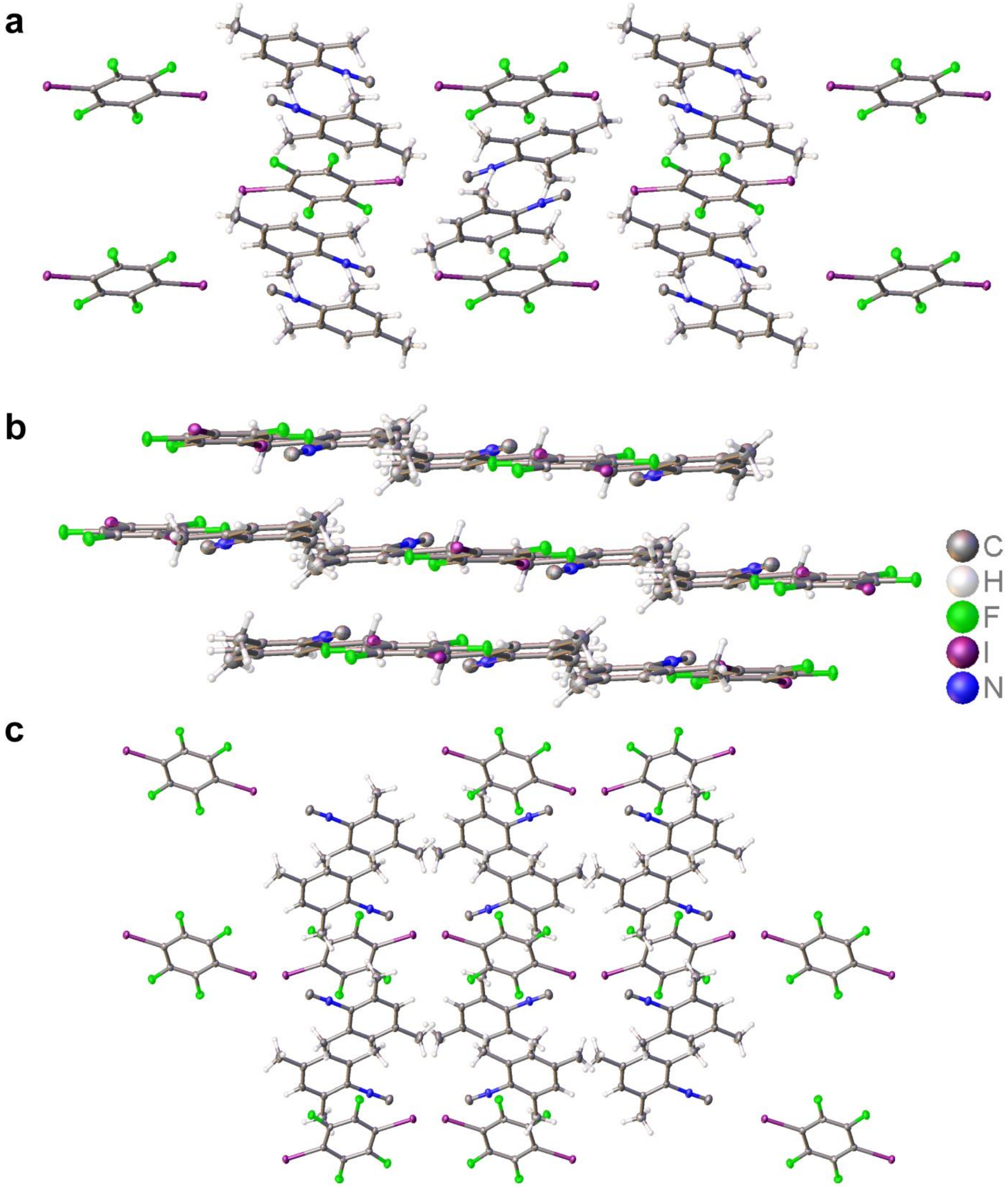

Supplementary Figure 5 Views along **a**, **b**, and **c** crystallographic axis of $(CNMes)_2$•1,4-FIB.

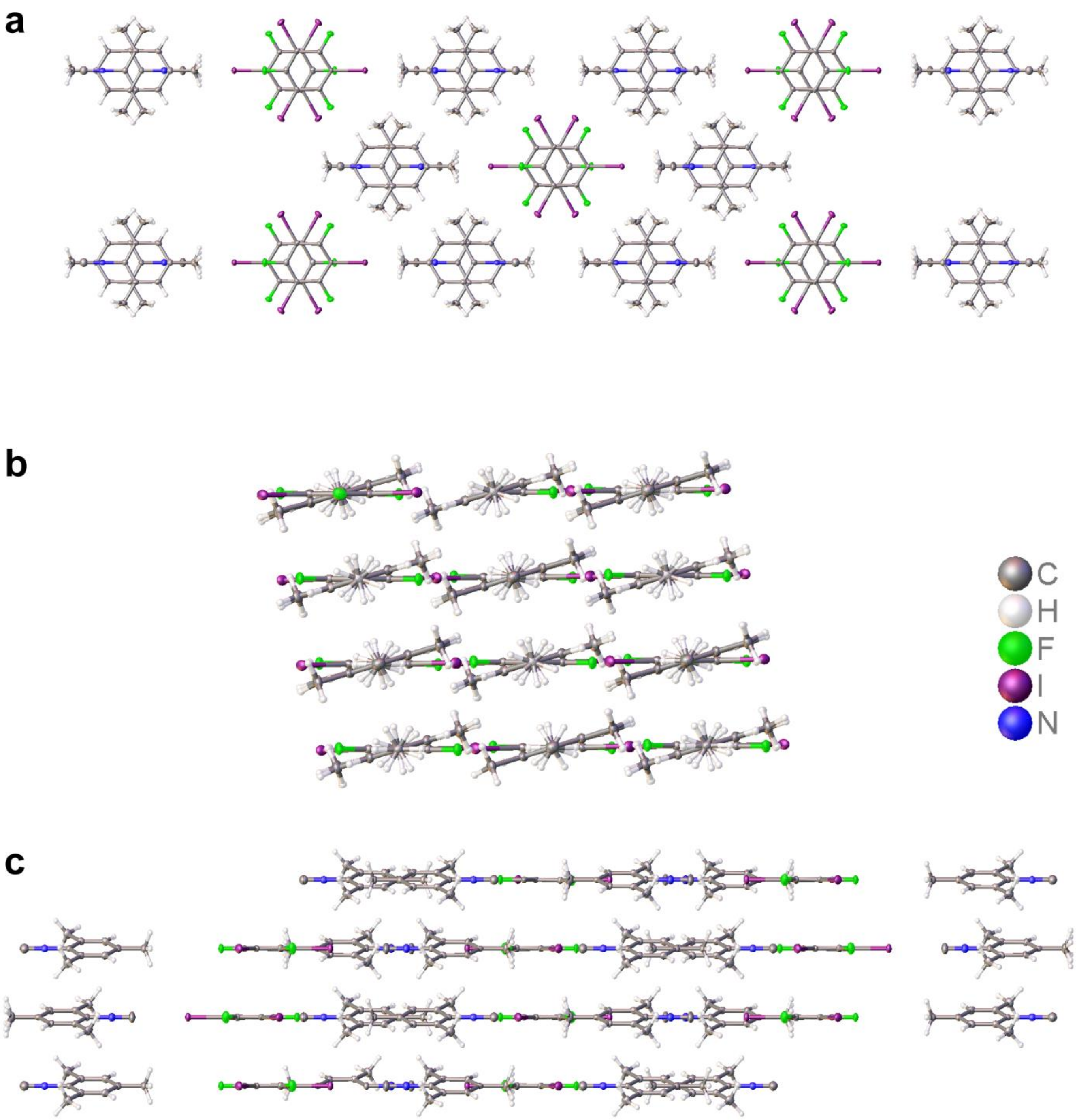

Supplementary Figure 6 Views along **a**, **b**, and **c** crystallographic axis of $(CNMes)_2$•1,3,5-FIB.

Supplementary Table 3 Verification of Hal•••C contacts involving isocyano carbon atom in CSD

CCDC code	Structure	Contact	*d*(Hal•••C), Å	Bondi vdW radii Σ	Bondi vdW radii %	∠(C–Hal•••C), (º)	∠(Hal•••CN), (º)
**BBZIC**	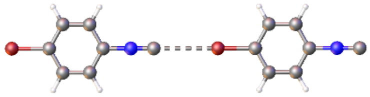	Br•••C	3.294(3)	3.55	93	180	180
**IBZICN**	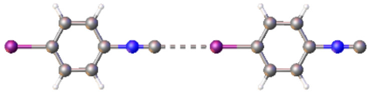	I•••C	3.217(5)	3.68	88	180	180
**FUGVAE**	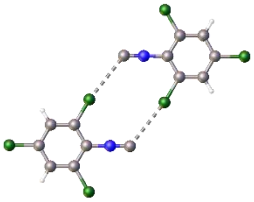	Cl•••C	3.245(4)	3.45	94	169.9(1)	127.4(2)
**MESRAG**	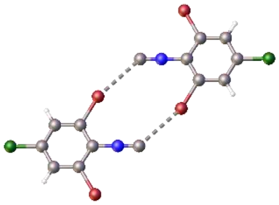	Br•••C	3.125(2)	3.55	88	163.66(6)	135.95(3)
**MESRIO**	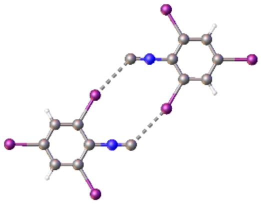	I•••C	3.106(2)	3.68	84	166.37(5)	134.18(3)
**TBZINT**	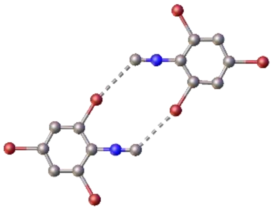	Br•••C	3.181(1)	3.55	90	165.54(1)	132.37(1)
**TBZINT01**			3.141(4)		88	165.41(1)	134.0(1)
**REMLING**	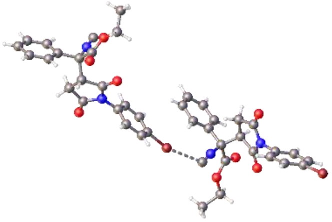	Br•••C	3.11(1)	3.55	88	162.8(4)	128.3(8)
**FOGFUD**	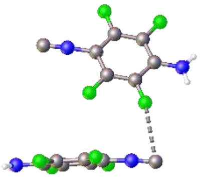	F•••C	3.166(2)	3.17	100	167.1(1)	76.4(1)

The structure **PEKWIN** was not considered in the search because of the misleading in the deposited structure – the original paper reports *p*-cyanobenzoic acid instead of *p*-isocyanobenzoic acid.[1]

Supplementary Table 4 Lengths for the selected covalent bonds in CNMes, the XB adducts, and iodoperfluorobenzenes

Structure	*d*(I–C), Å	*d*(C≡N), Å
CNMes	–	1.158(4)
(CNMes)•IPFB	2.096(5)	1.148(7)
IPFB (CCDC: ZAHGAQ)	2.077(4)	–
(CNMes)•1,2-FIB	2.098(4)	1.147(5)
	2.085(4)	
	2.095(4)	1.147(5)
	2.092(4)	
1,2-FIB (CCDC: WSBOR)	2.083(2)	–
	2.087(2)	–
$(CNMes)_2$•1,4-FIB	2.089(2)	1.157(3)
1,4-FIB (CCDC: ZZZAVM01)	2.075(1)	–
$(CNMes)_2$•1,3,5-FIB	2.096(4)	1.146(7)
	2.089(3)	1.156(6)
1,3,5-FIB (CCDC: UCEPEY)	2.069(7)	–
	2.070(6)	–
	2.082(7)	–

Identification of XB by solid-state ^{13}C NMR

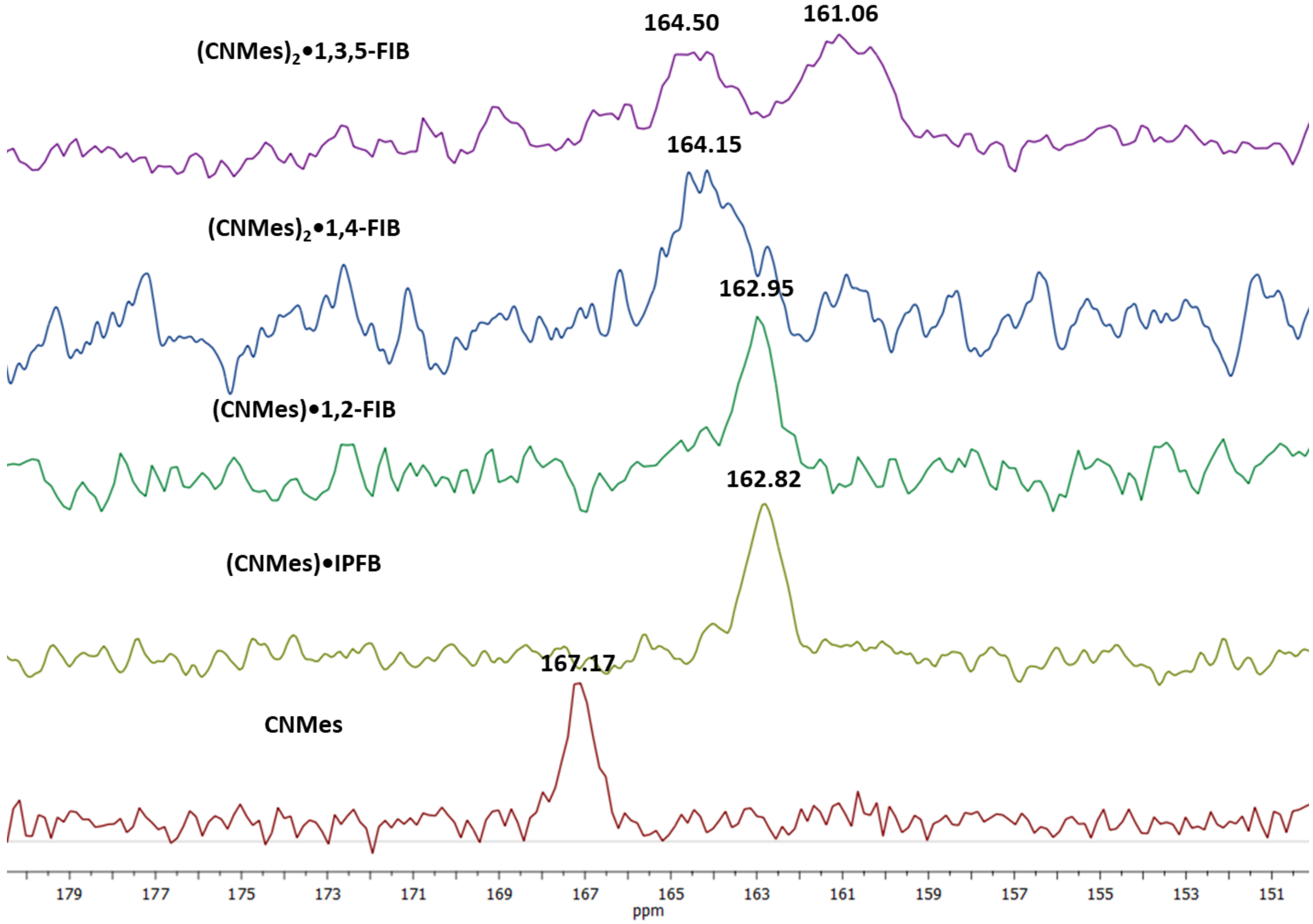

Supplementary Figure 7 Comparison of chemical shift of isocyano C signal in the solid-state ^{13}C CP/MAS NMR spectra for CNMes and its XB adducts

Supplementary Table 5 Values of the density of all electrons – ρ(r), Laplacian of electron density – $\nabla^2\rho(r)$, energy density – H_b, potential energy density – V(r), and Lagrangian kinetic energy – G(r) (a.u.) at the bond critical point (3, –1), corresponding to XBs in XRD and gas phase optimized equilibrium structures of XB adducts with CNMes, bond lengths – *d* (Å), Wiberg bond indexes (WI), as well as energies for this contacts E_{int} (kcal/mol), defined by different approaches

Contact	ρ(**r**)	$\nabla^2\rho(\mathbf{r})$	H_b	V(**r**)	G(**r**)	E_{int}^a	E_{int}^b	E_{int}^c	E_{int}^d	*d*	WI
(CNMes)•IPFB (XRD)											
I1D•••C1A	0.015	0.057	0.001	-0.007	0.011	2.2	3.0	3.0	4.6	3.134	0.07
C1A•••I1D	0.005	0.015	0.001	-0.002	0.003	0.6	0.8	0.9	1.3	3.831	0.01
I1D•••I1D	0.002	0.007	0.000	-0.001	0.001	0.3	0.3	0.4	0.4	4.641	0.00
(CNMes)•IPFB (gas phase)											
I1D•••C1A	0.021	0.078	0.001	-0.009	0.014	2.8	3.8	3.8	5.9	2.978	0.10
(CNMes)•1,2-FIB (XRD)											
I2D1•••I1D2	0.006	0.017	0.001	-0.003	0.003	0.9	0.8	1.3	1.3	4.040	0.01
I2D1•••I2D2	0.005	0.014	0.001	-0.002	0.003	0.6	0.8	0.9	1.3	4.261	0.00
I2D2•••I1D1	0.006	0.019	0.001	-0.003	0.004	0.9	1.1	1.3	1.7	3.975	0.02
I2D2•••C1A1	0.005	0.014	0.001	-0.002	0.003	0.6	0.8	0.9	1.3	3.817	0.01
I1D1•••C1A1	0.019	0.071	0.001	-0.009	0.013	2.8	3.5	3.8	5.5	3.029	0.08
$(CNMes)_2$•1,4-FIB (XRD)											
I1D•••C1A	0.016	0.058	0.001	-0.007	0.011	2.2	3.0	3.0	4.6	3.121	0.07
$(CNMes)_2$•1,3,5-FIB (XRD)											
I1D•••C1A1	0.014	0.052	0.001	-0.006	0.010	1.9	2.7	2.6	4.2	3.186	0.06
I2D•••C1A2	0.009	0.033	0.001	-0.004	0.006	1.3	1.6	1.7	2.5	3.334	0.03

[a] $E_{int} = -V(\mathbf{r})/2$;[10] [b] $E_{int} = 0.429G(\mathbf{r})$;[11] [c] $E_{int} = 0.68(-V(\mathbf{r}))$;[12] [d] $E_{int} = 0.67G(\mathbf{r})$;[12]

Supplementary Table 6 Vertical and adiabatic dissociation energies for gas phase optimized structures of (CNMes)•IPFB adduct

Approach for energy estimation	Total electronic energies, a.u. XB acceptor	XB donor	Adduct	Dissociation energy, kcal/mol
Vertical	–72.8133367752	–165.999178377	–238.824557361	7.6
Adiabatic	–72.8139284512	–165.999683890	–238.824557361	6.9

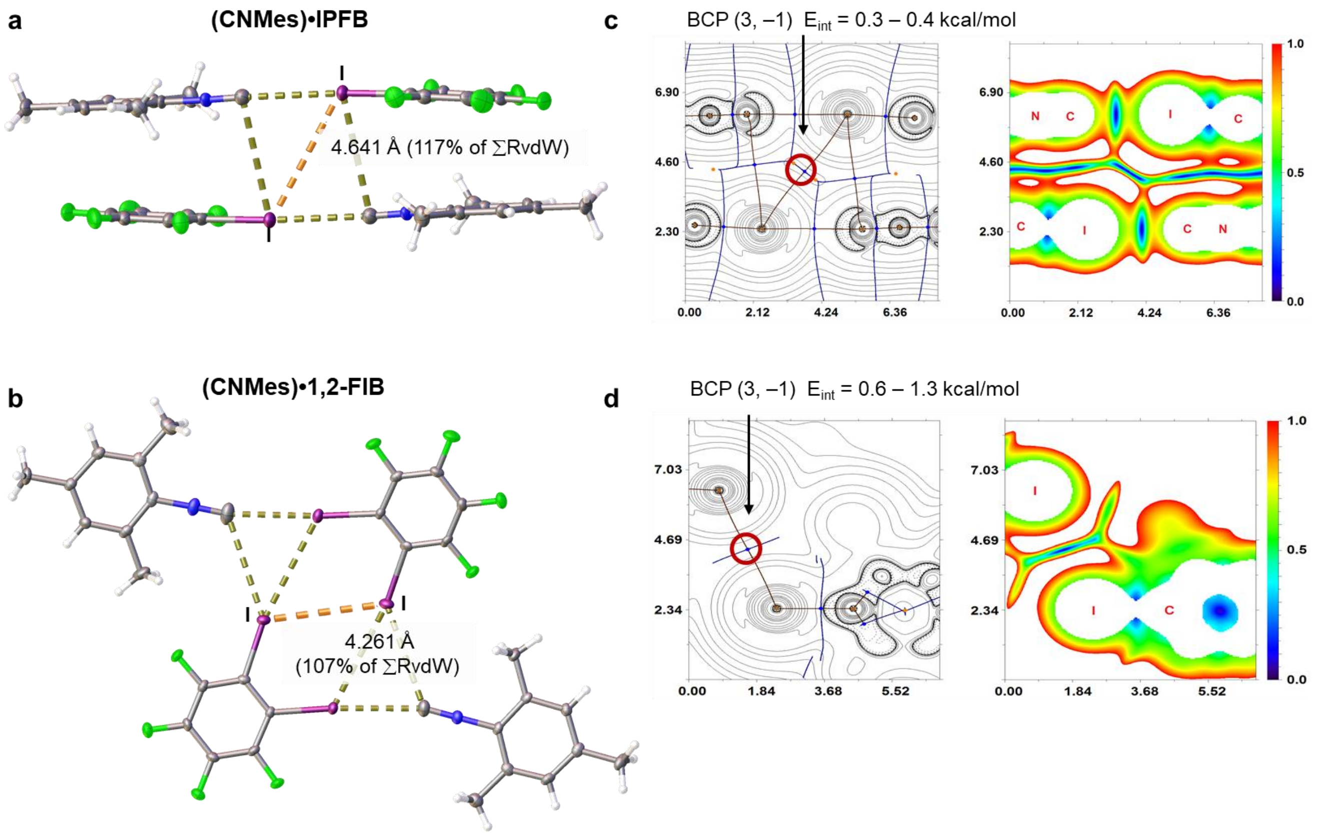

Supplementary Figure 8 Additional type I I•••I XBs in the structures of (CNMes)•IPFB and (CNMes)•1,2-FIB: **a**, **b** contacts in the crystal structures (orange dotted line) and **c**, **d** contour line diagrams of the Laplacian distribution $\nabla^2\rho(r)$, bond paths and selected zero-flux surfaces and RDG isosurfaces

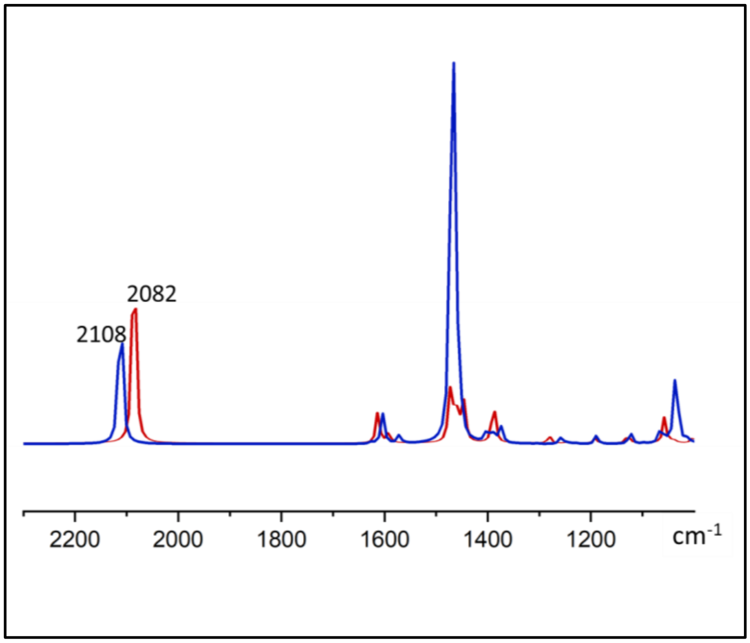

Supplementary Figure 9 Calculated IR spectra for optimized structures of free CNMes (red) and (CNMes)•IPFB adduct (blue)

ESP calculations. To get a deeper insight into the XB acceptor ability of isocyanide CNMes and compared it with other abundant types of XB acceptors, we performed the electrostatic surface potential (ESP) calculations for mesitylisocyanide (CNMes), mesitylnitrile (NCMes), 2,4,6-trimethylpyridine (2,4,6-Me_3Py), 2,4,6-trimethylpyridine *N*-oxide (2,4,6-$Me_3Py^+O^-$), and dimethylimidazolyl based *N*-heterocyclic carbene (IMe) model structures (**Supplementary Figure 9**).The full geometry optimization of these model structures been carried out at the DFT level of theory using the M06-2X functional[6] and CEP-121G basis sets[7,8] with the help of the Gaussian-09 program package[9] whereas The ESP for the all model structures was plotted using the Chemcraft program.[17] Based on the values of minimum of ESP, the isocyanides (–41 kcal/mol) should have comparable XB acceptor ability with the isomeric nitrile NCMes (–44 kcal/mol) or 2,4,6-trimethylpyridine (–45 kcal/mol). At the same time, they all are inferior to aromatic *N*-oxides (–53 kcal/mol) and also to the strongly donating push-push type *N*-heterocyclic carbenes (–54 kcal/mol).

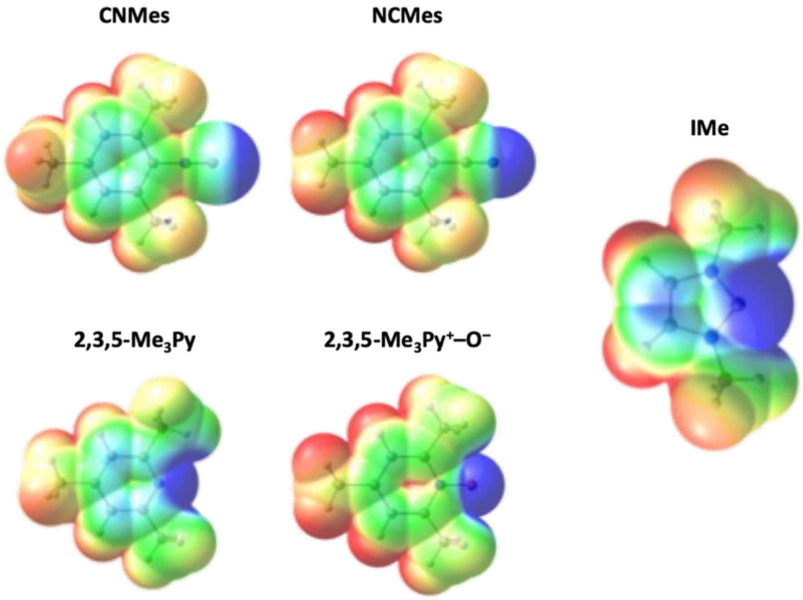

Supplementary Figure 10. Distribution of electrostatic potential $V_S(\mathbf{r})$ calculated on the 0.001 a.u. molecular surfaces for the optimized equilibrium geometries of CNMes, NCMes, 2,4,6-MePy, 2,4,6-$Me_3Py^+O^-$, and IMe model structures at the M06-2X/CEP-121G level of theory. From red to blue, the electrostatic potential is becoming increasingly negative; the color scheme is taken from Politzer's work.[18]

GC-MS odor measurements

Supplementary Table 7 GS-MS measurements of relative isocyanide concentration in gas phase above the crystal phase for CNMes and its adducts

Sample	Probe	Peak area (•10^{-4})	CNMes peak areas ratio (free $CNMes_{(av.)}$/Adduct)
CNMes	1	520	–
	2	480	
	3	389	
	4	455	
	5	368	
	**av.**	**443±51**	
(CNMes)•IPFB	1	147	3.0
	2	154	2.9
	3	150	3.0
	4	130	3.4
	5	136	3.3
	**av.**	**143±8**	**3.1±0.2**
(CNMes)•1,2-FIB	1	8	57
	2	15	31
	3	12	38
	4	8	57
	5	9	48
	**av.**	**10±2.5**	**46±10**
$(CNMes)_2$•1,4-FIB	1	137	3.2
	2	149	3.0
	3	156	2.8
	4	112	4.0
	5	112	3.9
	**av.**	**133±17**	**3.4±0.5**
$(CNMes)_2$•1,3,5-FIB	1	71	6.3
	2	88	5.1
	3	55	8.1
	4	46	9.7
	5	80	5.6
	**av.**	**68±14**	**6.9±1.6**

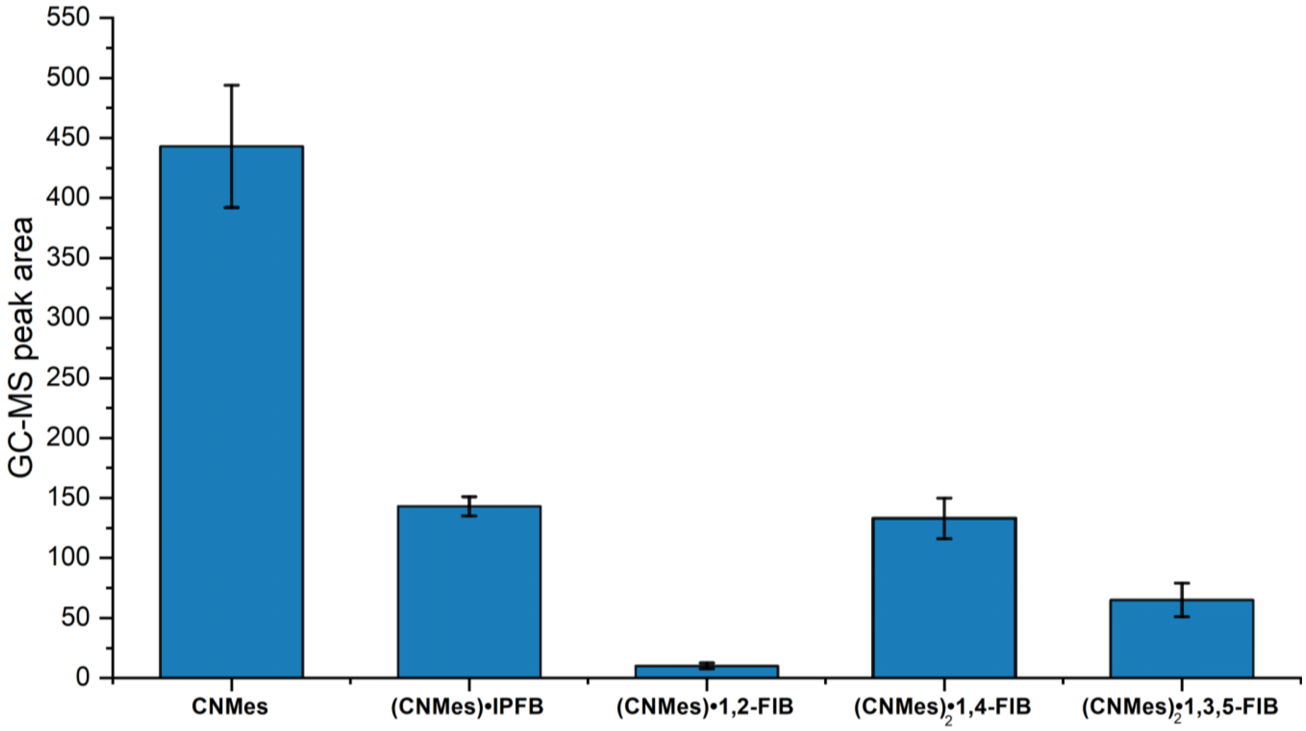

Supplementary Figure 11 Average GS-MS peak areas representing the isocyanide concentration in gas phase above the crystal phase for CNMes and its adducts. The error bars represent a standard deviation. The data are taken from **Supplementary Table 7**.

SUPPLEMENTARY METHODS

Reactivity tests

To demonstrate the potential laboratory usage of isocyanide XB adducts we compared the reactivity of the (CNMes)•IPFB adduct, taken as a model system, with the parent CNMes in some most common transformations that include ligation to such metal centers as Au^{I}, Pt^{II}, and Pd^{II} and the multi-component Ugi reaction (**Supplementary Figure 12**).

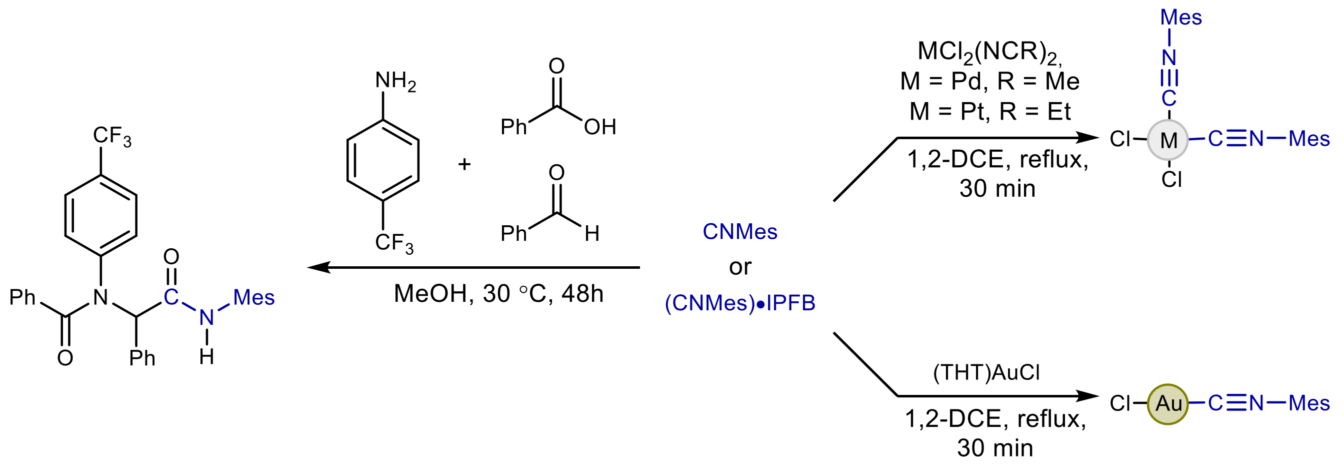

Supplementary Figure 12 Reaction of the (CNMes)•IPFB adduct or the free isocyanide with organic and inorganic substrates

Materials and Instrumentation. Solvents and organic reagents were obtained from commercial sources and used as received. The AuCl(THT),[19] $PdCl_2(MeCN)_2$,[20] and $PtCl_2(EtCN)_2$[21] complexes were synthesized according literature procedures. ^{1}H (400 MHz), ^{13}C (101 MHz), and ^{19}F (376 MHz) NMR spectra were acquired on a Bruker Avance 400 MHz spectrometer in CDCl3 at 298K. Melting points were determined in capillaries with a Stuart SMP 30 apparatus. The mass spectra were obtained on a Bruker micrOTOF spectrometer equipped with electrospray ionization (ESI) source; MeOH was used as a solvent. The instrument was operated at positive ion mode using an m/z range of 50−3000. The capillary voltage of the ion source was set at −4500 V (ESI+) and the capillary exit at ±(70−150) V. The nebulizer

gas pressure was 0.4 bar and drying gas flow 4.0 L/min. Infrared spectra were recorded on a Shimadzu IRAffinity-1S FT-IR spectrometer (4000−400 cm^{-1}) in KBr pellets.

Complexation. The gold(I)[22], palladium(II)[23] and platinum(II)[24] mesityl isocyanide complexes were synthesized according a modified literature procedure.[21] Solid CNMes (20 mg, 0.14 mmol) or (CNMes)•IPFB adduct (60 mg, 0.14 mmol) was dissolved in 1 mL of 1,2-DCE and added dropwise to a solution or suspension of the corresponding complex (AuCl(THT) 44 mg, 0.14 mmol; $PdCl_2(MeCN)_2$ 18 mg, 0.07 mmol; $PtCl_2(EtCN)_2$ 26 mg, 0.07 mmol) in 1,2-DCE (2 mL) placed in 10-mL round-bottom flask, and the reaction mixture was then refluxed for ca. 30 min. The reaction mixtures became gradually pale yellow and in the cases of palladium(II) and platinum(II) the products precipitated as pale yellow powders. The solvent was removed under low pressure. To remove residual IPFB the resulting solid was washed with petroleum ether (3×2 mL). The isolated yields are given for the reactions with (CNMes)•IPFB adduct. The obtained NMR data are consistent with the literature results.[22-24]

AuCl(CNMes)[22] (48 mg, 92%) ^1H NMR (400 MHz, $CDCl_3$) δ 6.95 (s, 2H), 2.39 (s, 6H), 2.32 (s, 3H).

$PdCl_2(CNMes)_2$[23] (30 mg, 93%) ^1H NMR (400 MHz,$CDCl_3$, *cis*/*trans*-isomers – ratio 4/1): δ 6.96 (s, 4H, *cis*-isomer), 6.93 (s, 4H, *trans*-isomer), 2.43 (s, 12H, *cis*/*trans*-isomers), 2.32 (s, 6H, *cis*-isomer), 2.31 (s, 6H, *trans*-isomer).

$PtCl_2(CNMes)_2$[24] (36 mg, 94%) ^1H NMR (400 MHz, $CDCl_3$, *cis*-isomer) δ 6.96 (s, 4H), 2.42 (s, 12H), 2.32 (s, 6H).

Ugi reaction. The Ugi reaction were carried out in accordance with the next modified literature procedure.[25] A solution of *p*-trifluoromethylaniline (23 mg, 0.14 mmol) and benzaldehyde (15 mg, 0.14 mmol) in 3 mL of MeOH was added to solid CNMes (20 mg, 0.14 mmol) or (CNMes)•IPFB adduct (60 mg, 0.14 mmol) and benzoic acid (25 mg, 0.2 mmol) placed in 10-mL round-bottom flask. Then the reaction mixture was stirred at 30 °C for 48 h. The solvent was removed under low pressure. The crude product was purified by flash column chromatography (eluent 80:20 hexane/EtOAc). To remove residual IPFB the resulting solid was washed with petroleum ether (2×2 mL). The product was obtained as a white solid. The isolated yield is given for the reaction with (CNMes)•IPFB adduct.

N-(2-(mesitylamino)-2-oxo-1-phenylethyl)-N-(4-(trifluoromethyl)phenyl)benzamide (49 mg, 69%). Mp = 173.0 – 173.5 °C. HR-ESIMS: for $[M + H]^+$ *m/z* 517.2097 ($C_{31}H_{28}F_3N_2O_2^+$ calcd), 517.2101 (found); for $[M + Na]^+$ *m/z* 539.1917 ($C_{31}H_{27}F_3N_2O_2Na^+$ calcd), 539.1920 (found). FTIR (KBr, selected bands, cm^{-1}): 3258, 3038, 2919, 1655, 1636, 1611, 1531, 1481, 1325. ^1H NMR (400 MHz, $CDCl_3$), δ: 7.40 – 7.33 (m, 2H), 7.32 – 7.27 (m, 5H), 7.25 – 7.19 (m, 3H), 7.18 – 7.11 (m, 4H), 7.10 (s, 1H), 6.88 (s, 2H), 6.39 (s, z1H), 2.25 (s, 3H), 2.19 (s, 6H). ^{13}C NMR (101 MHz, $CDCl_3$) δ 171.12 (1C), 168.19 (1C), 144.29 (1C), 136.93 (1C), 135.46 (1C), 135.20 (1C), 133.96 (1C), 130.99 (2C), 130.69 (1C), 130.21 (2C), 129.75 (1C), 128.89 (q, 2C, $J^3_{C–F}$ = 13 Hz), 128.82 (2C), 128.68 (2C), 128.32 (2C), 127.78 (2C), 123.58 (q, 1C, $J^1_{C–F}$ = 272 Hz), 122.22 (q, 2C, $J^4_{C–F}$ = 3.5 Hz), 66.34 (1C), 20.80 (1C), 18.34 (2C). Signal of *ipso*-carbon at CF_3-group is not found presumably because of low intensity and overlapping with over signals. ^{19}F NMR (376 MHz, $CDCl_3$): –62.62.

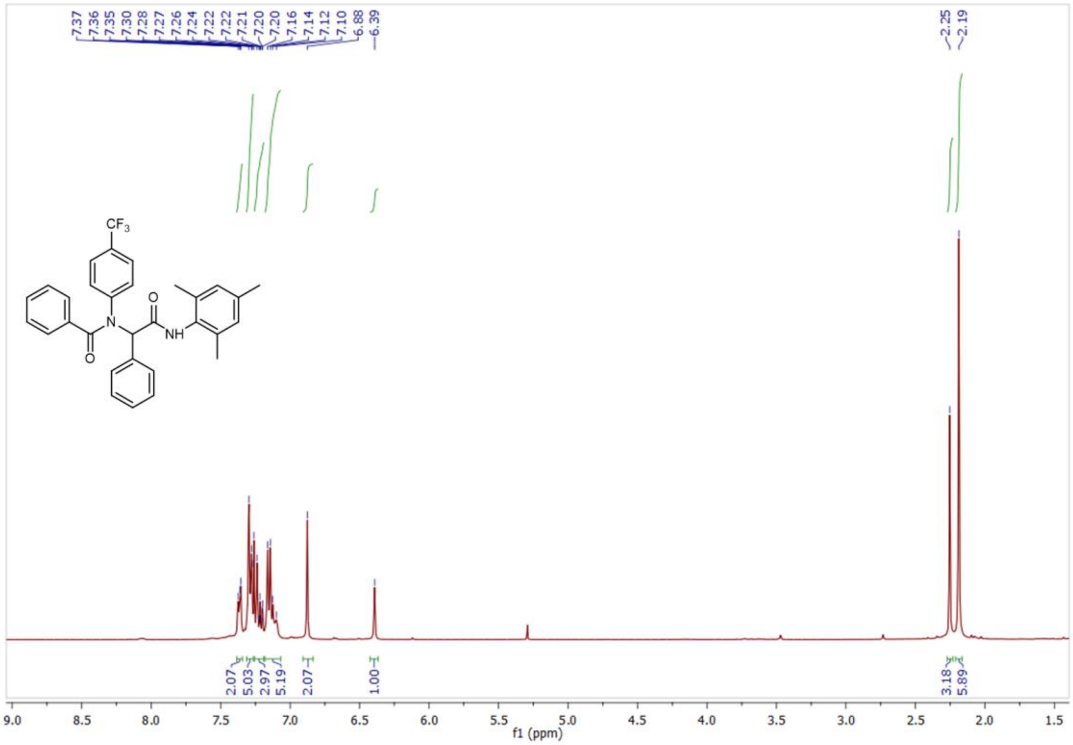

Supplementary Figure 13 ^{1}H NMR spectrum of the Ugi reaction product in $CDCl_3$ at 298K

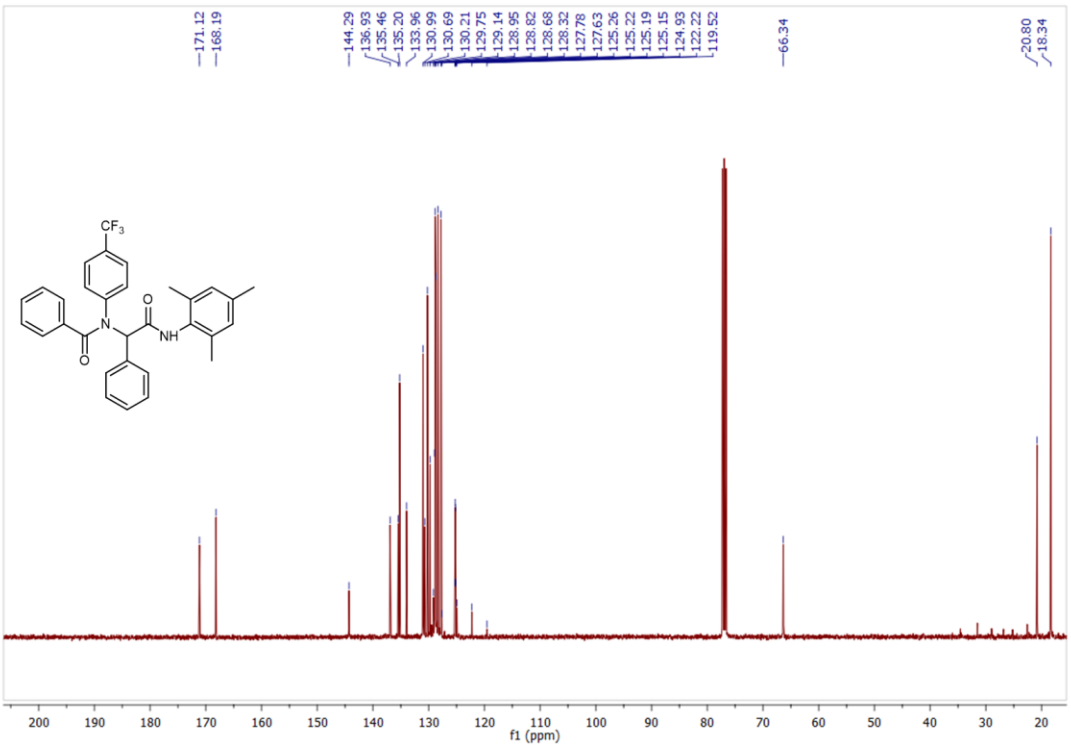

Supplementary Figure 14 ^{13}C NMR spectrum of the Ugi reaction product in $CDCl_3$ at 298K

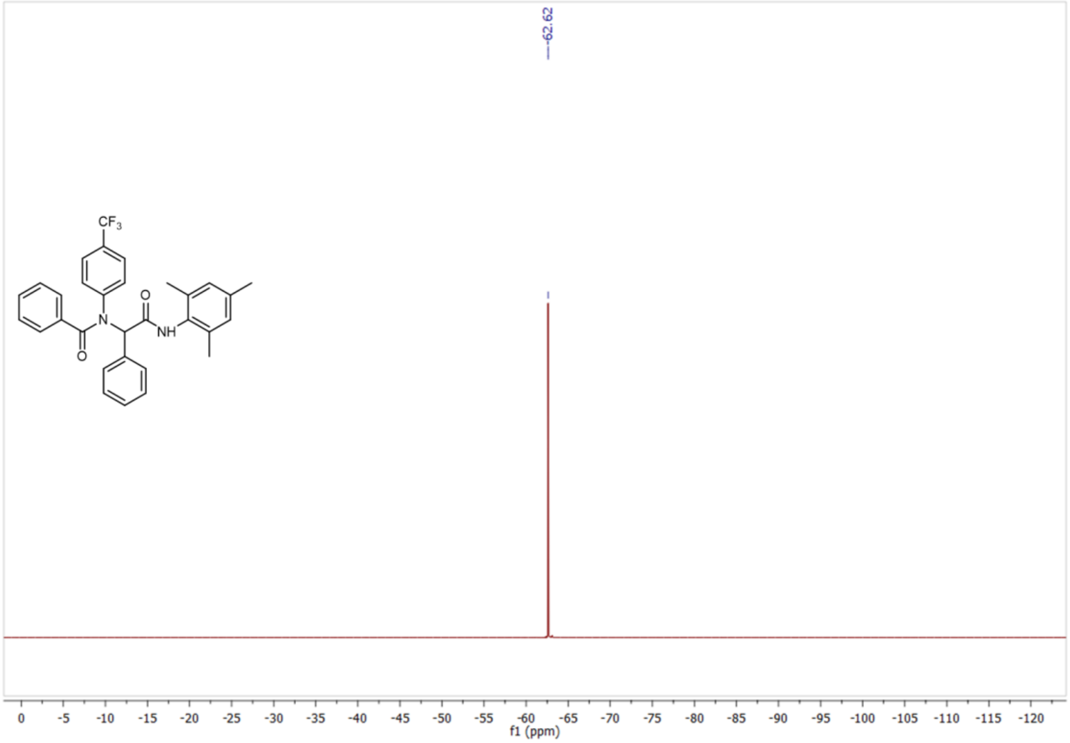

Supplementary Figure 15 ^{19}F NMR spectrum of the Ugi reaction product in $CDCl_3$ at 298K

Single-crystal X-ray diffraction

Supplementary Table 8 Crystal data and structure refinement for the XB adducts with CNMes

Identification code	(CNMes)•IPFB	(CNMes)•IPFB 200K	(CNMes)•1,2-FIB	$(CNMes)_2$•1,4-FIB	$(CNMes)_2$•1,4-FIB 200K	$(CNMes)_2$•1,3,5-FIB
CCDC Code	1957698	1981526	1957699	1957700	1981527	1957701
Empirical formula	$C_{16}H_{11}F_5IN$	$C_{16}H_{11}F_5IN$	$C_{32}H_{22}F_8I_4N_2$	$C_{13}H_{11}F_2IN$	$C_{13}H_{11}F_2IN$	$C_{26}H_{22}F_3I_3N_2$
Formula weight	439.17	439.16	1094.11	346.13	346.13	800.15
Temperature/K	122.4(7)	200.00(10)	100.01(10)	100.01(10)	200.00(10)	100.01(10)
Crystal system	triclinic	triclinic	triclinic	monoclinic	monoclinic	monoclinic
Space group	P-1	P-1	P-1	$P2_1/c$	$P2_1/c$	I2/a
a/Å	8.0389(3)	7.9778(5)	11.6289(4)	9.5236(5)	9.58710(10)	7.1214(5)
b/Å	9.0006(3)	9.6129(6)	12.0046(4)	16.6800(7)	16.7370(2)	31.801(2)
c/Å	11.5201(3)	11.1713(7)	13.0323(5)	8.3936(4)	8.49750(10)	12.1531(6)
α/°	81.512(2)	85.635(5)	96.184(3)	90	90	90
β/°	73.403(3)	71.940(6)	100.277(3)	105.843(5)	105.9000(10)	101.815(6)
γ/°	87.161(3)	88.981(5)	105.500(3)	90	90	90
Volume/Å^3	790.04(5)	812.14(9)	1701.69(11)	1282.70(11)	1311.34(3)	2694.0(3)
Z	2	2	2	4	4	4
ρ_{calc}g/cm^3	1.8460	1.796	2.135	1.792	1.753	1.973
μ/mm^{-1}	16.409	2.019	3.732	2.498	19.231	3.518
F(000)	425.3	424.0	1024.0	668.0	668.0	1512.0
Crystal size/mm^3	0.1 × 0.1 × 0.1	0.1 × 0.1 × 0.1	0.1 × 0.1 × 0.1	0.1 × 0.1 × 0.1	0.1 × 0.1 × 0.1	0.1 × 0.1 × 0.1
Radiation	Cu Kα (λ = 1.54184)	MoKα (λ = 0.71073)	MoKα (λ = 0.71073)	MoKα (λ = 0.71073)	CuKα (λ = 1.54184)	MoKα (λ = 0.71073)
2Θ range for data collection/°	8.08 to 145.32	5.37 to 64.52	5.864 to 63.074	5.606 to 61.85	9.592 to 140.82	5.984 to 61.928
Index ranges	-9 ≤ h ≤ 8, -11 ≤ k ≤ 11, -14 ≤ l ≤ 13	-10 ≤ h ≤ 11, -13 ≤ k ≤ 14, -10 ≤ l ≤ 16	-16 ≤ h ≤ 16, -17 ≤ k ≤ 17, -17 ≤ l ≤ 17	-13 ≤ h ≤ 13, -23 ≤ k ≤ 24, -11 ≤ l ≤ 11	-11 ≤ h ≤ 11, -18 ≤ k ≤ 20, -10 ≤ l ≤ 10	-10 ≤ h ≤ 5, -45 ≤ k ≤ 17, -14 ≤ l ≤ 16
Reflections collected	9131	8793	33134	14679	7323	7606
Independent reflections	3087 [R_{int} = 0.0696, R_{sigma} = 0.0525]	5149 [Rint = 0.0217, Rsigma = 0.0390]	9933 [R_{int} = 0.0430, R_{sigma} = 0.0516]	3707 [R_{int} = 0.0328, R_{sigma} = 0.0318]	2509 [Rint = 0.0332, Rsigma = 0.0317]	3747 [R_{int} = 0.0281, R_{sigma} = 0.0455]
Data/restraints/parameters	3087/0/211	5149/0/211	9933/0/421	3707/0/157	2509/0/157	3747/0/165
Goodness-of-fit on F^2	1.038	1.055	1.095	1.081	1.068	1.061
Final R indexes [I>=2σ (I)]	R_1 = 0.0439, wR_2 = 0.1178	R1 = 0.0362, wR2 = 0.0719	R_1 = 0.0339, wR_2 = 0.0562	R_1 = 0.0250, wR_2 = 0.0476	R1 = 0.0324, wR2 = 0.0801	R_1 = 0.0314, wR_2 = 0.0598
Final R indexes [all data]	R_1 = 0.0453, wR_2 = 0.1192	R1 = 0.0495, wR2 = 0.0793	R_1 = 0.0596, wR_2 = 0.0664	R_1 = 0.0354, wR_2 = 0.0522	R1 = 0.0344, wR2 = 0.0820	R_1 = 0.0409, wR_2 = 0.0651
Largest diff. peak/hole / e Å^{-3}	1.88/-1.75	0.51/-0.54	1.33/-0.80	1.08/-0.49	0.45/-1.30	0.97/-1.15

Powder X-ray diffraction

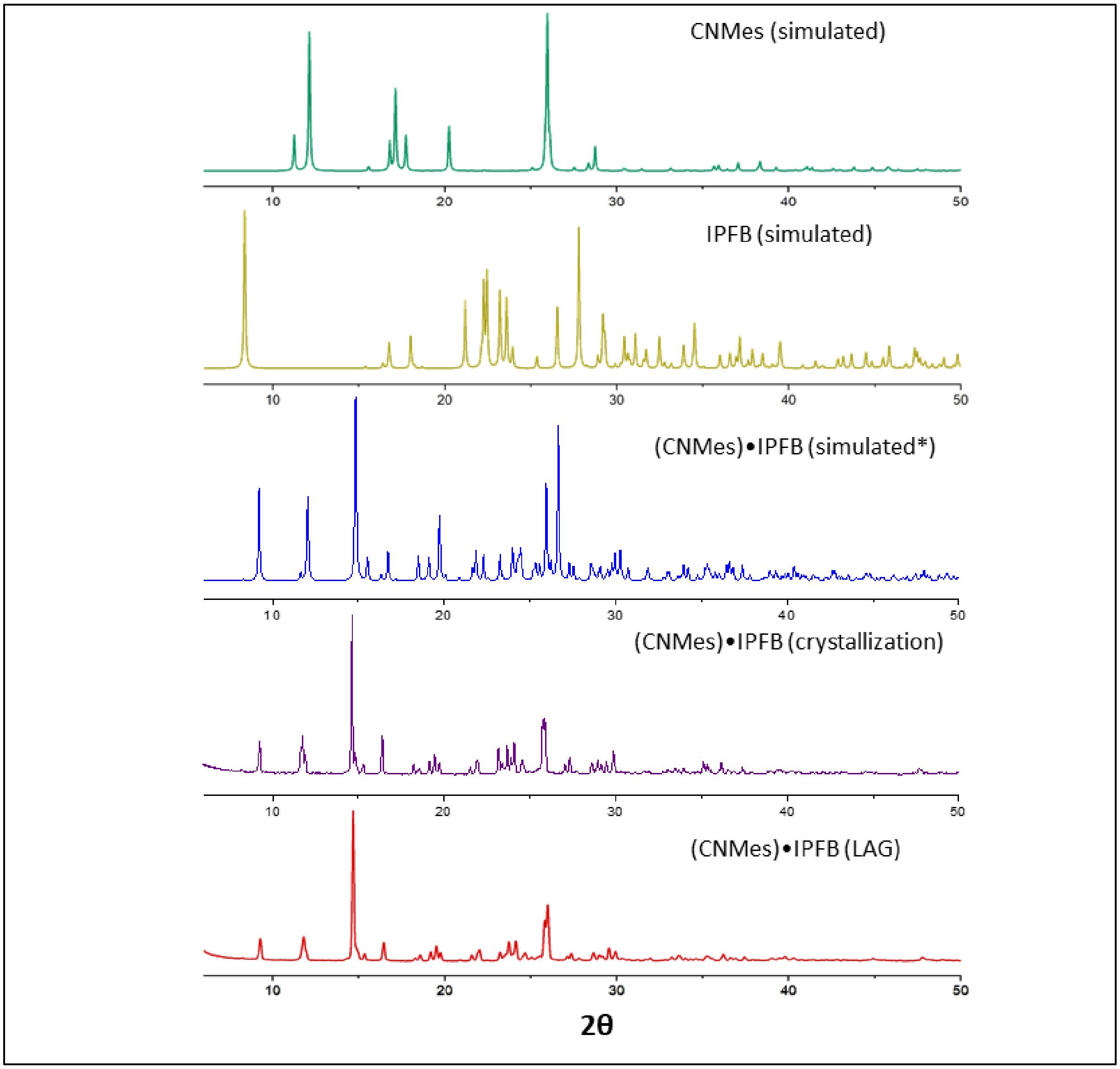

Supplementary Figure 16 X-ray powder diffraction patterns: CNMes, IPFB, (CNMes)•IPFB adduct (simulated from the crystal structures; *XRD measurements performed at 200K) and (CNMes)•IPFB adduct obtained by LAG approach (experimental)

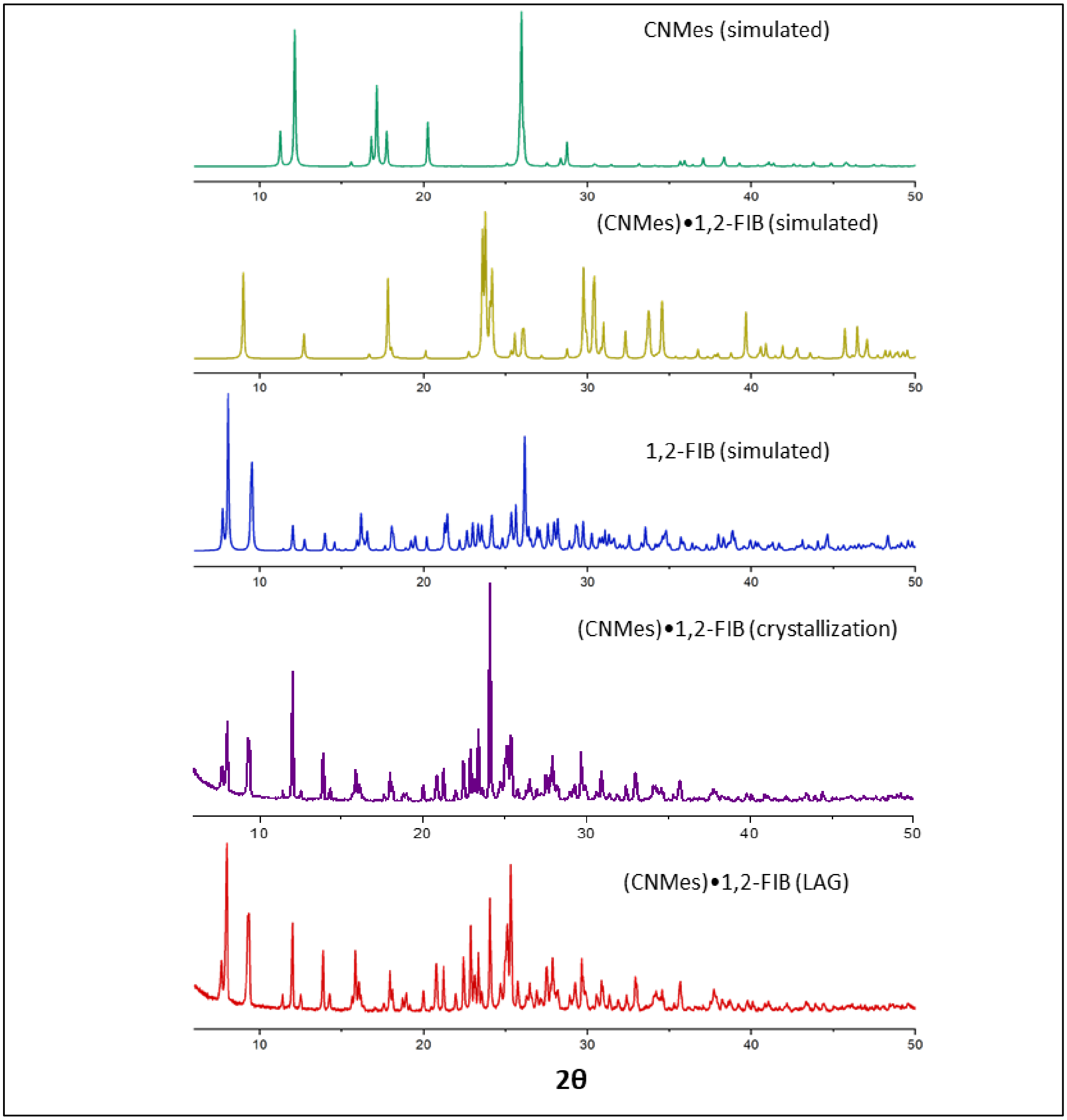

Supplementary Figure 17 X-ray powder diffraction patterns: CNMes, 1,2-FIB, (CNMes)•1,2-FIB adduct (simulated from the crystal structures) and (CNMes)•1,2-FIB adduct obtained by LAG approach (experimental)

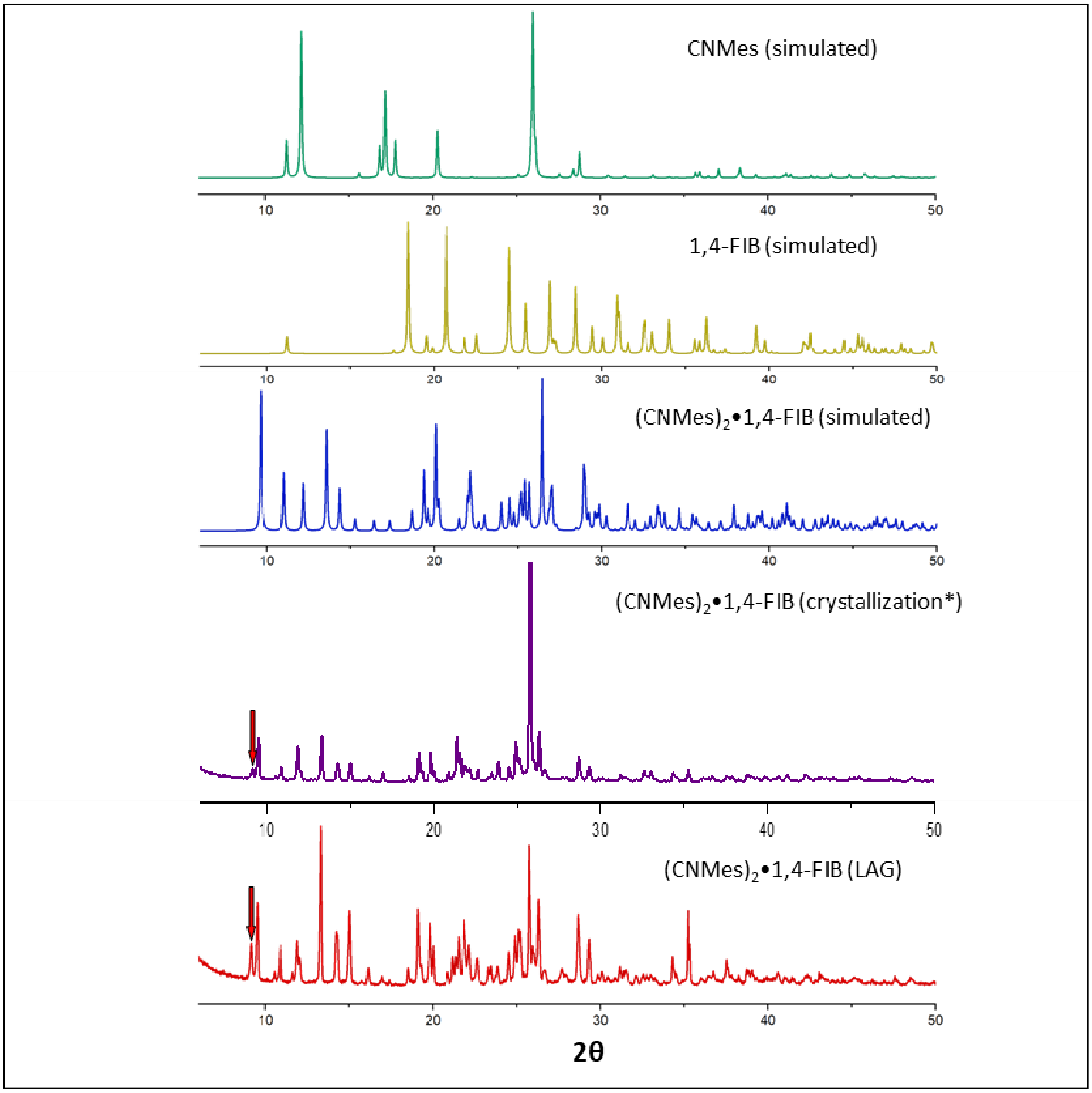

Supplementary Figure 18 X-ray powder diffraction patterns: CNMes, 1,4-FIB, $(CNMes)_2$•1,4-FIB adduct (*simulated from the crystal structures XRD measurements performed at 200K) and $(CNMes)_2$• 1,4-FIB adduct obtained by LAG approach (experimental). The red arrow represents an impurity, which could be either another polymorphic modification of the (CNMes)2•1,4-FIB adduct, or a minor impurity.

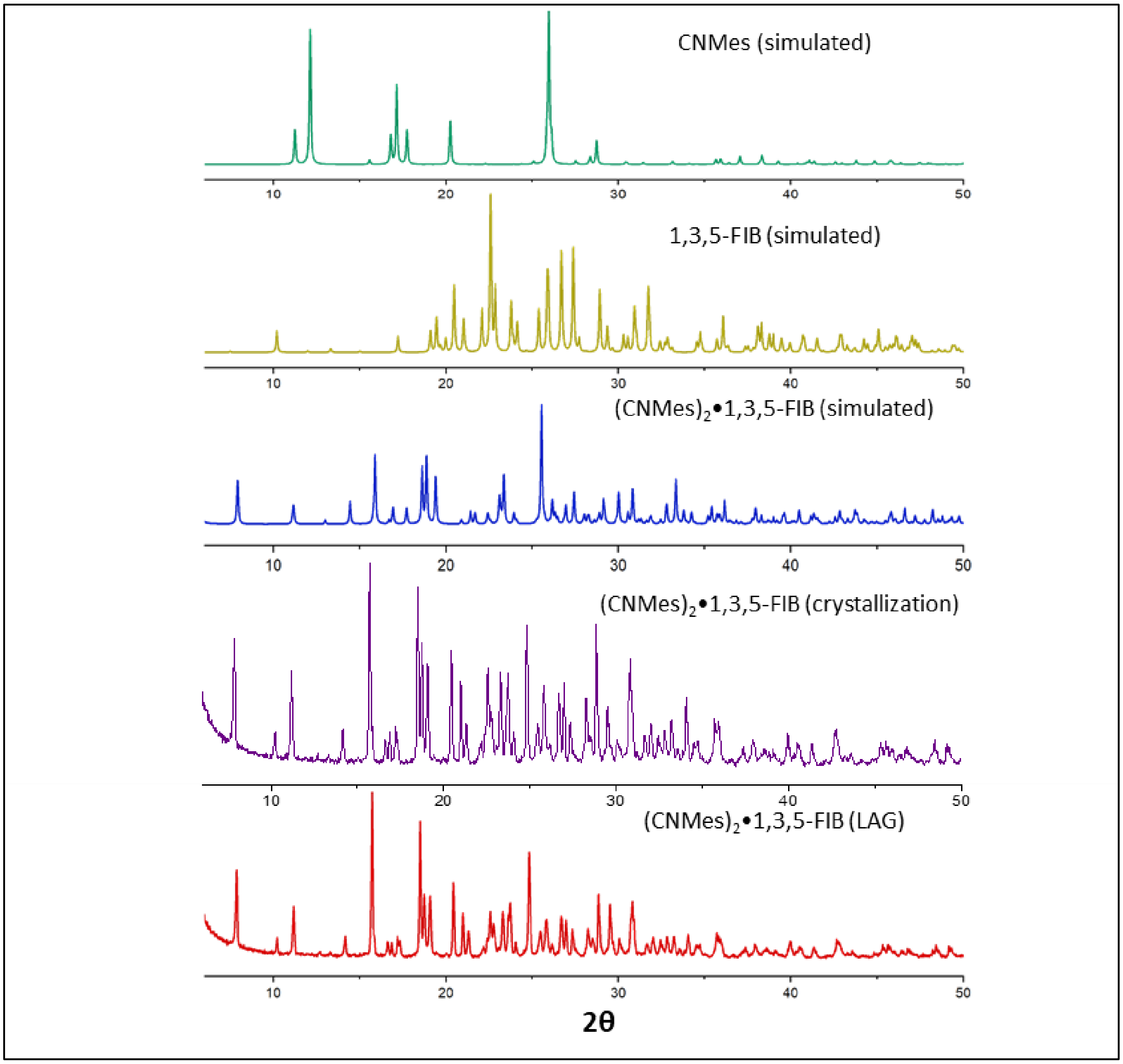

Supplementary Figure 19 X-ray powder diffraction patterns: CNMes, 1,3,5-FIB, $(CNMes)_2$•1,3,5-FIB adduct (simulated from the crystal structures) and $(CNMes)_2$• 1,3,5-FIB adduct obtained by LAG approach (experimental)

Solid-state ^{13}C NMR data

Supplementary Table 9 Experimental solid-state ^{13}C NMR conditions			
Sample	Relaxation delay	Number of scans	Spinning speed
CNMes	2	512	10
(CNMes)•IPFB (crystallization)	5	2048	6
(CNMes)•IPFB (LAG)	2	2048	14
(CNMes)•1,2-FIB (crystallization)	2	4096	10
(CNMes)•1,2-FIB (LAG)	2	2048	15
$(CNMes)_2$•1,4-FIB (crystallization)	2	4096	12.5
$(CNMes)_2$•1,4-FIB (LAG)	2	4096	15
$(CNMes)_2$•1,3,5-FIB (crystallization)	2	4096	10
$(CNMes)_2$•1,3,5-FIB (LAG)	2	4096	15

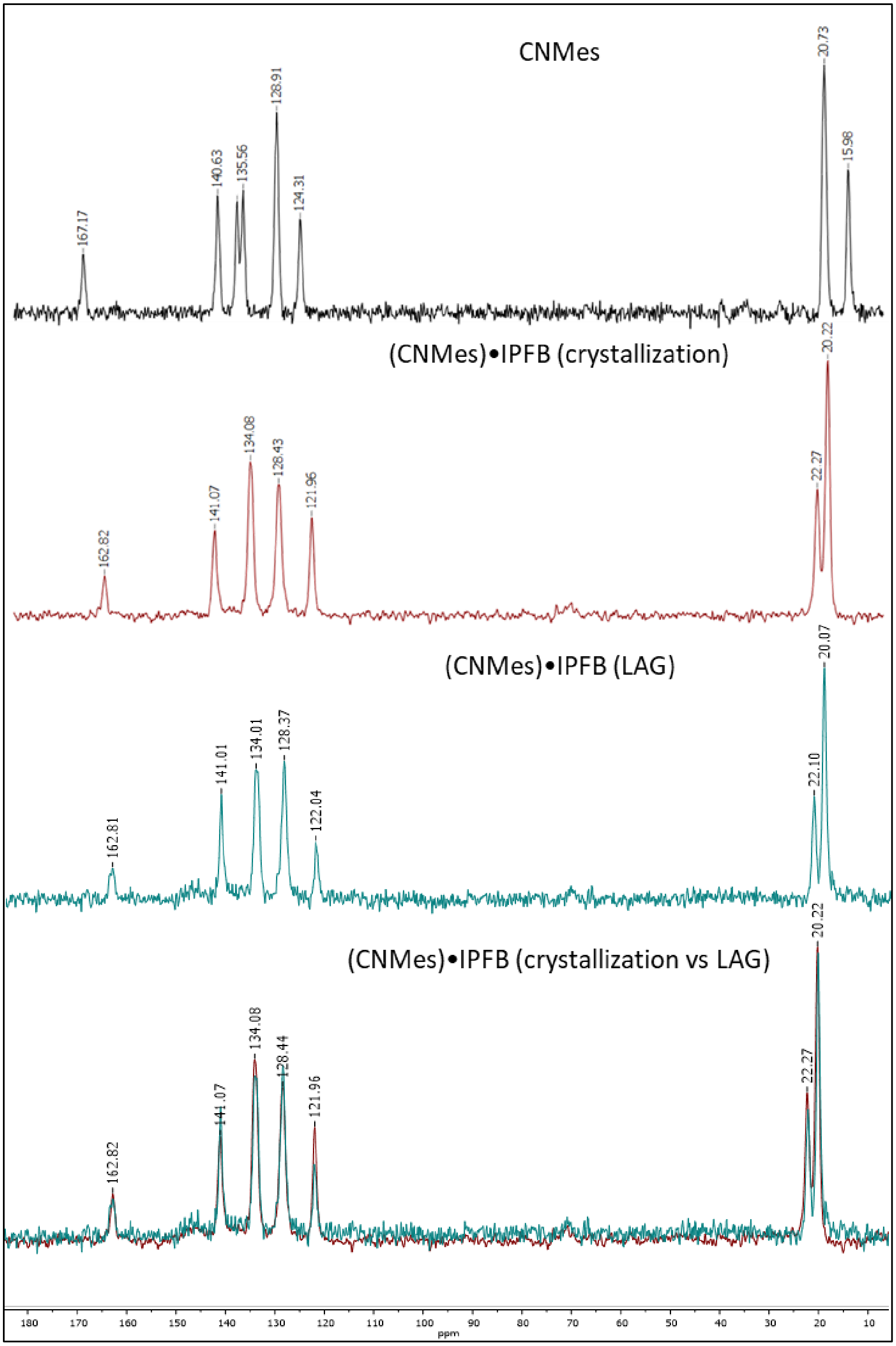

Supplementary Figure 20 Solid-state ^{13}C CP/MAS NMR spectra for CNMes and (CNMes)•IPFB adduct obtained by two approaches

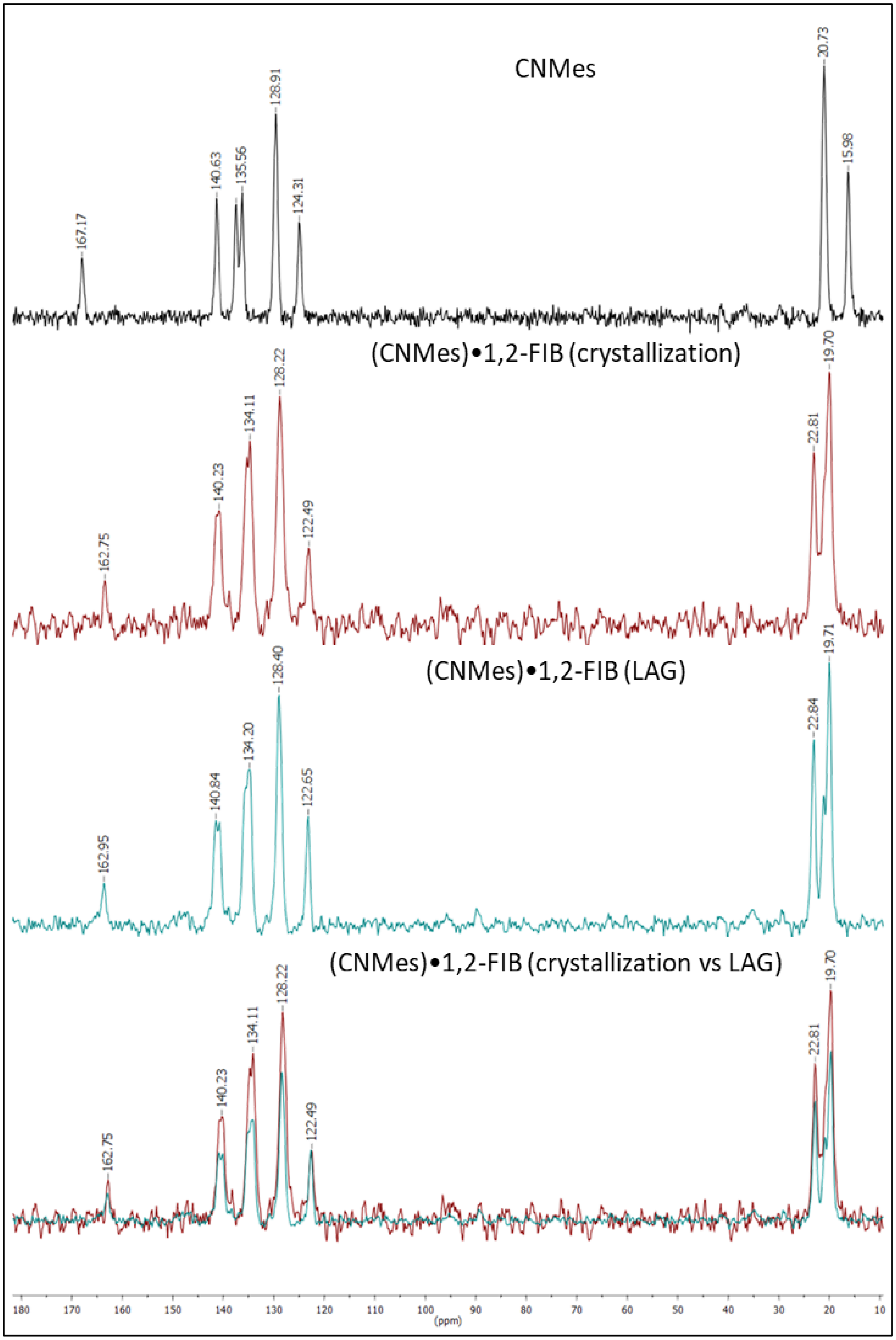

Supplementary Figure 21 Solid-state ^{13}C CP/MAS NMR spectra for CNMes and (CNMes)•1,2-FIB adduct obtained by two approaches

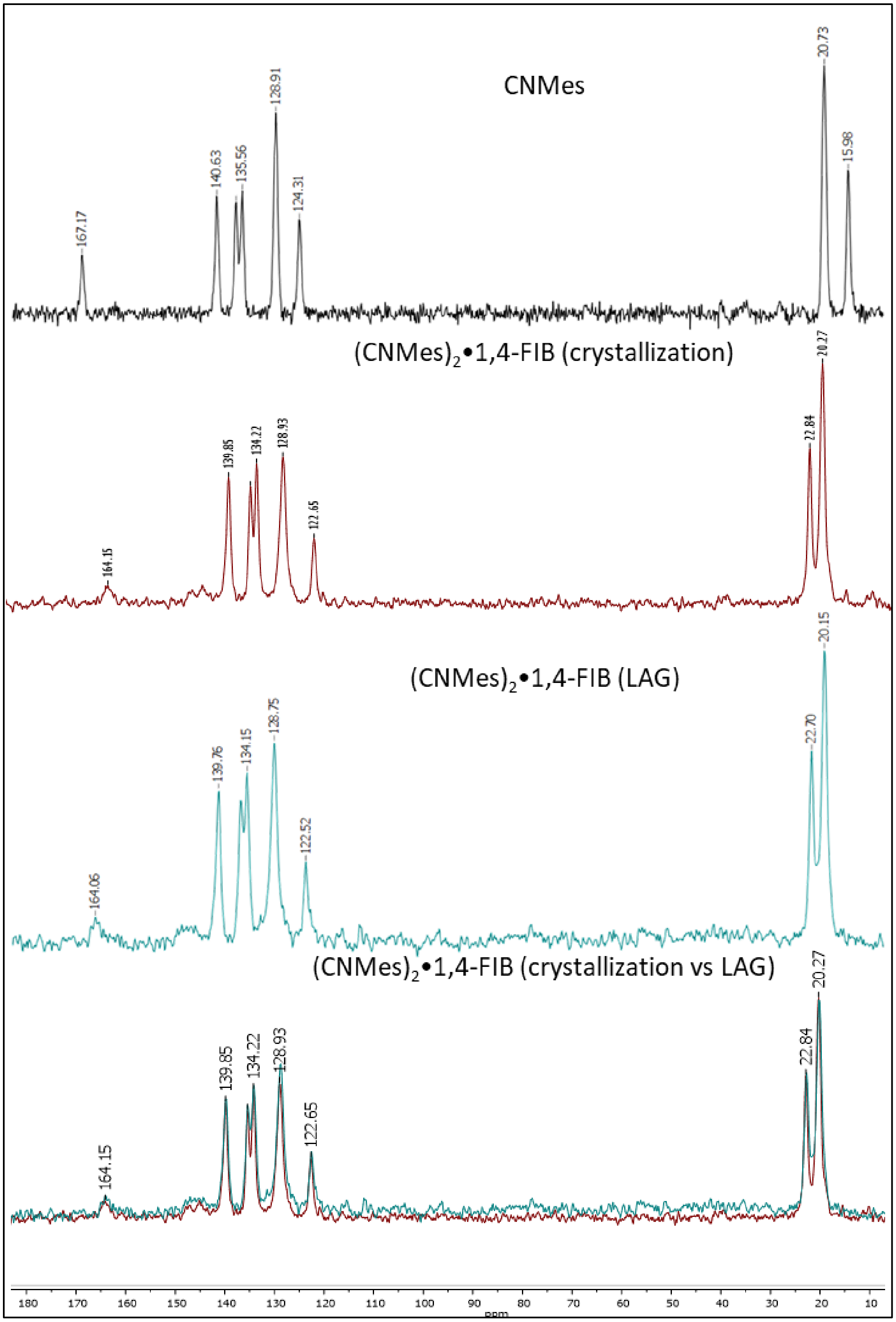

Supplementary Figure 22 Solid-state ^{13}C CP/MAS NMR spectra for CNMes and $(CNMes)_2$•1,4-FIB adduct obtained by two approaches

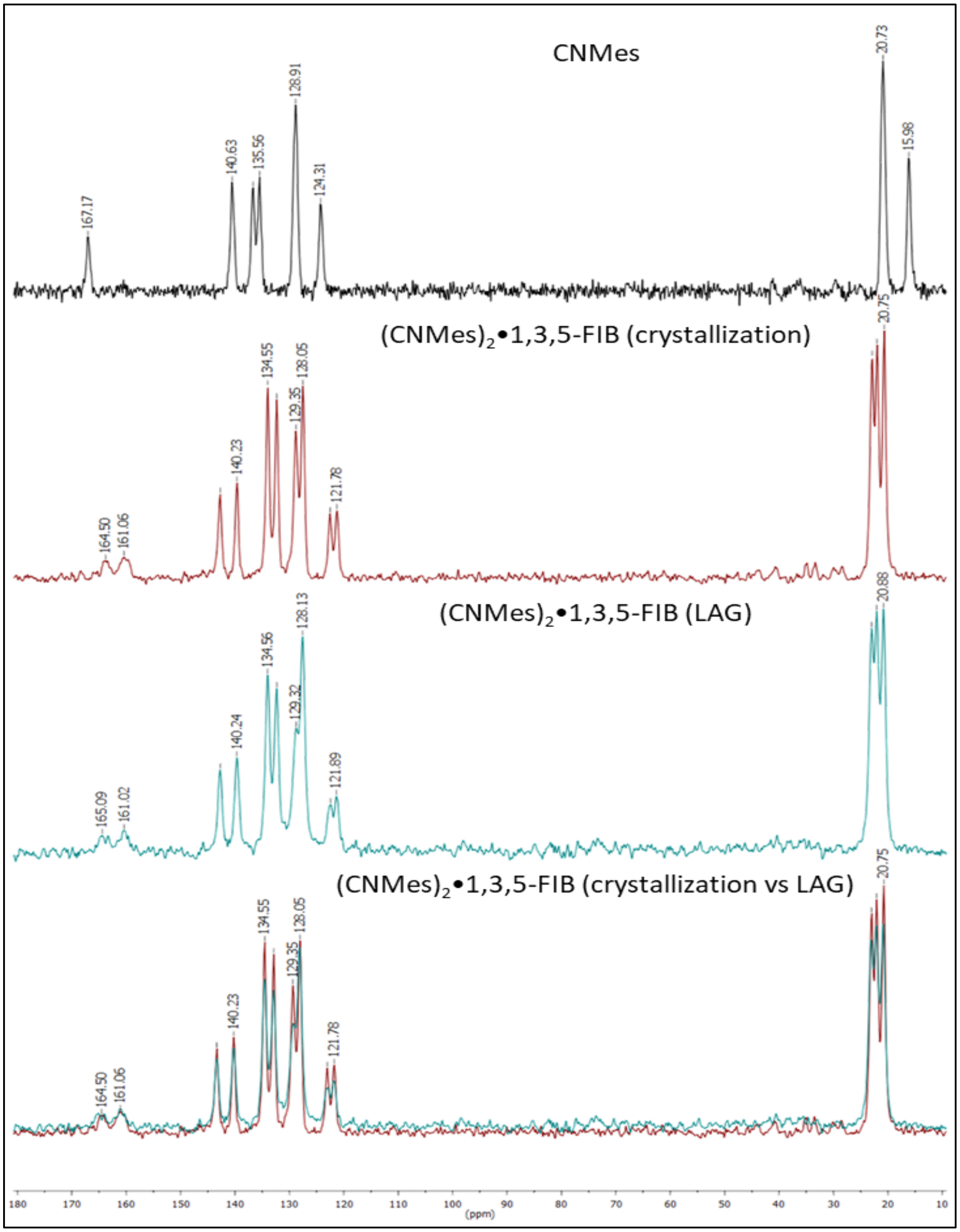

Supplementary Figure 23 Solid-state ^{13}C CP/MAS NMR spectra for CNMes and $(CNMes)_2$•1,3,5-FIB adduct obtained by two approaches

FTIR spectra

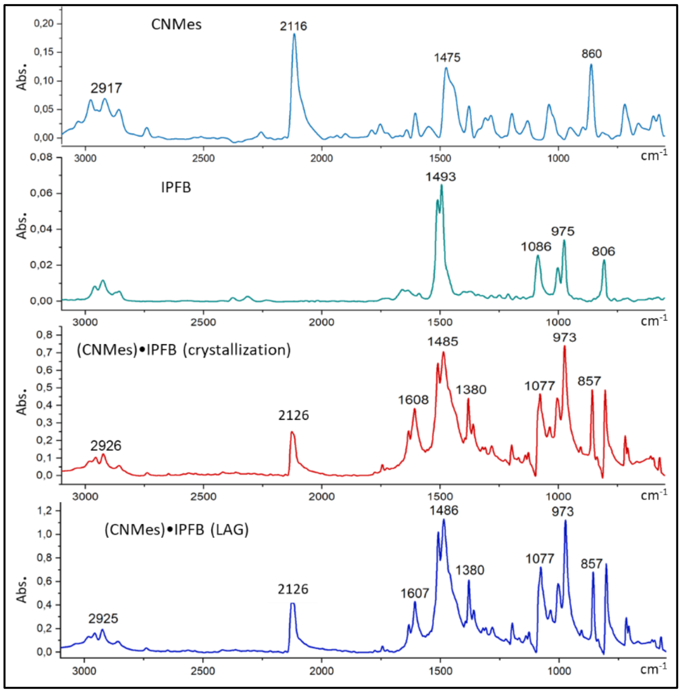

Supplementary Figure 24 FTIR spectra (KBr) for CNMes, IPFB, and (CNMes)•IPFB adduct obtained by two approaches

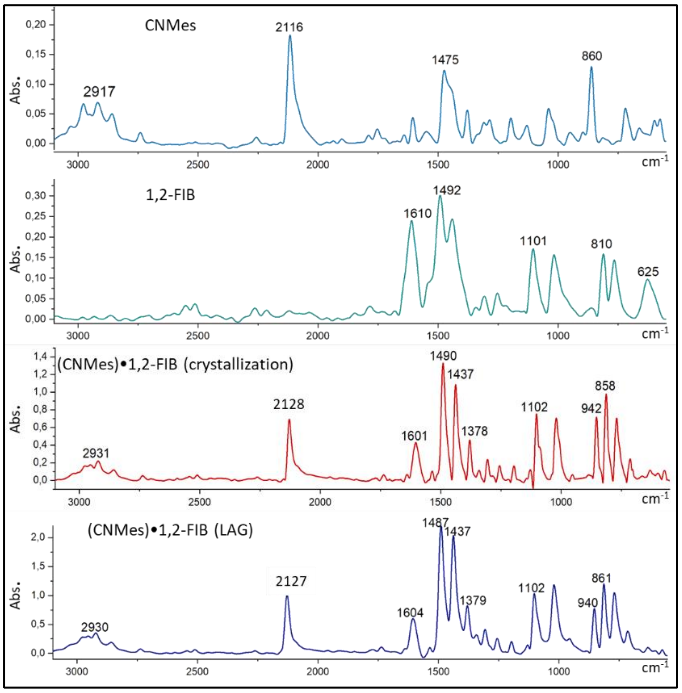

Supplementary Figure 25 FTIR spectra (KBr) for CNMes, 1,2-FIB, and (CNMes)•1,2-FIB adduct obtained by two approaches

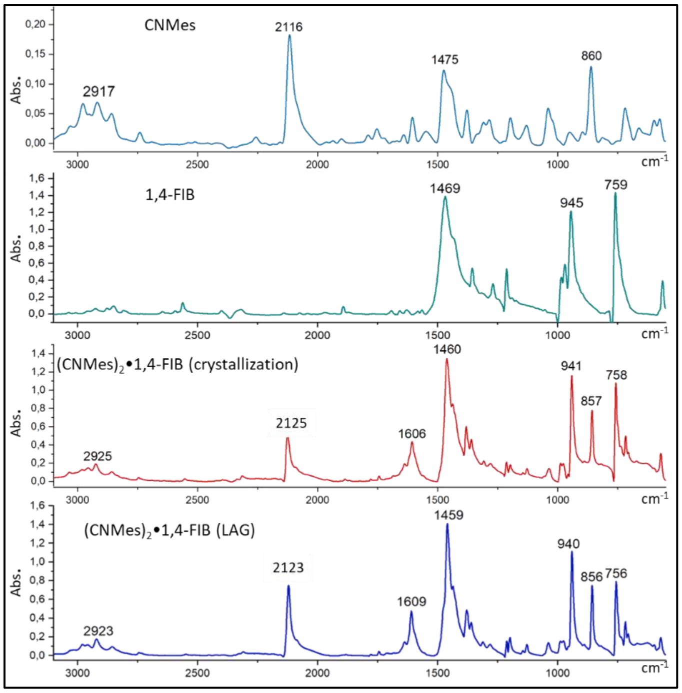

Supplementary Figure 26 FTIR spectra (KBr) for CNMes, 1,4-FIB, and $(CNMes)_2$•1,4-FIB adduct obtained by two approaches

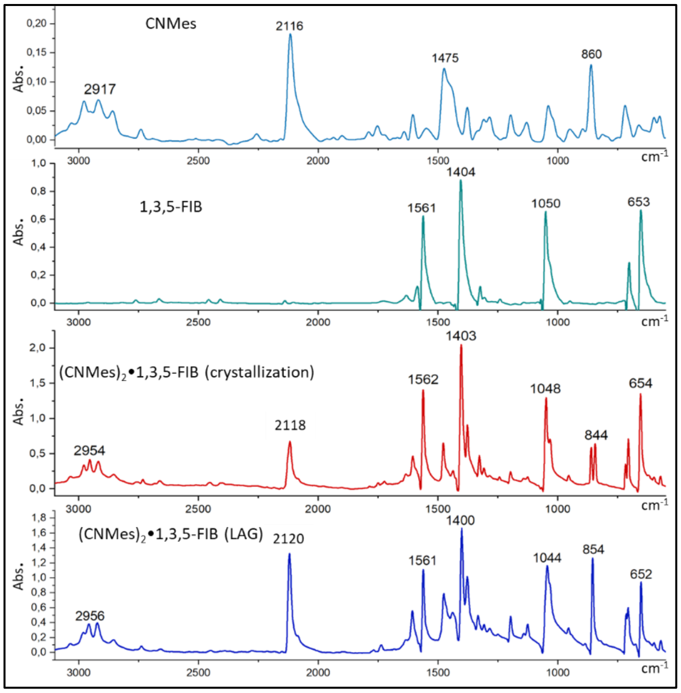

Supplementary Figure 27 FTIR spectra (KBr) for CNMes, 1,3,5-FIB, and $(CNMes)_2$•1,3,5-FIB adduct obtained by two approaches

Thermal analysis. In accord with the obtained thermogravimetric data (**Supplementary Figure 28–30**), the XB adducts with solid 1,2-, 1,4-, 1,3,5-FIBs exhibit a similar or even better thermal stability as the parent isocyanide and their decomposition starts in the range 110–125 °C (1% mass loss). In the adduct with liquid IPFB, the mass loss starts at a lower temperature (75 °C) and then continues with a smaller slope, what could suggest the elimination of IPFB from the crystal. Additionally, the formation of XB with CNMes leads to the higher melting point temperatures of the adducts than of the parent isocyanide, IPFB, and 1,2-FIB, but lower than of free 1,4- and 1,3,5-FIBs (**Supplementary Table 10**).

Supplementary Table 10 Melting points (°C) of XB adducts with CNMes and free CNMes

Sample	Adduct	XB donor	CNMes
(CNMes)•IPFB	43.3–43.7	–29[26]	40.0–41.0
(CNMes)•1,2-FIB	73.5–75.0	49–50[27]	
$(CNMes)_2$•1,4-FIB	83.5–84.5	108[28]	
$(CNMes)_2$•1,3,5-FIB	66.5–67.5	153[29]	

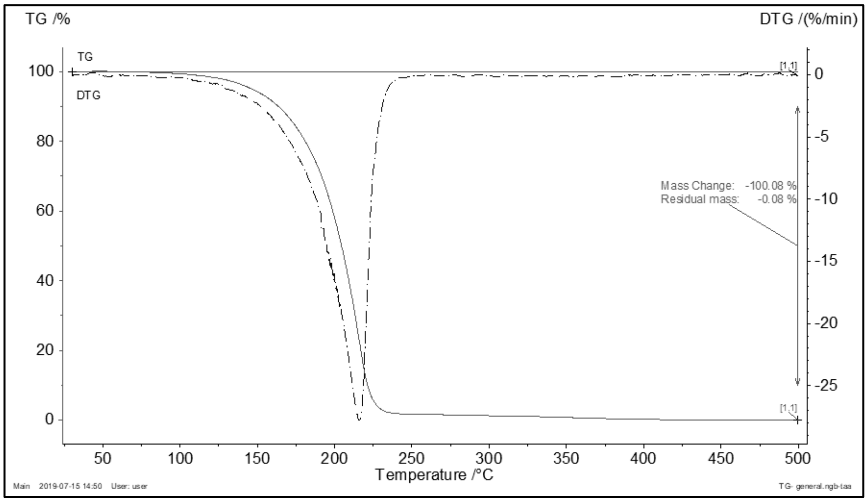

Supplementary Figure 28 TG/DTG curves of free CNMes

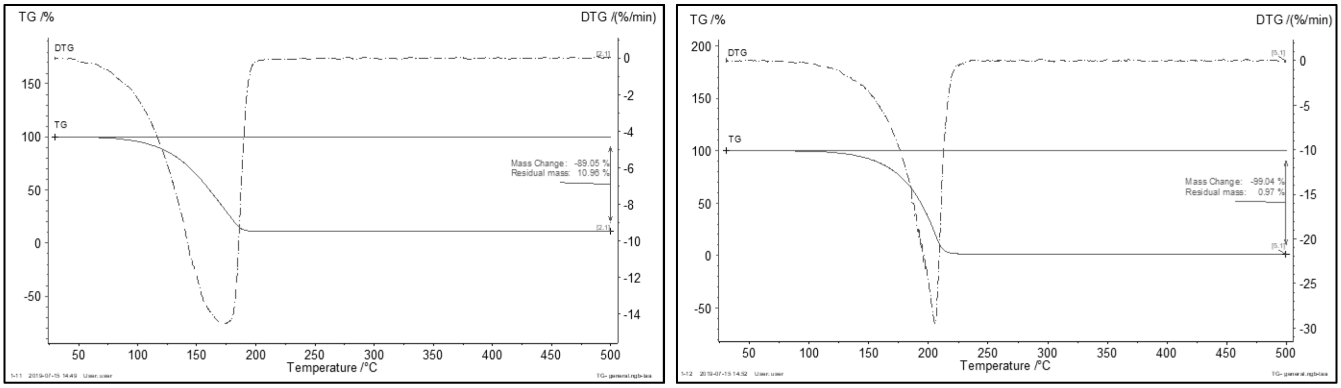

Supplementary Figure 29 TG/DTG curves of (CNMes)•IPFB (right) and (CNMes)•1,2-FIB (left) adducts

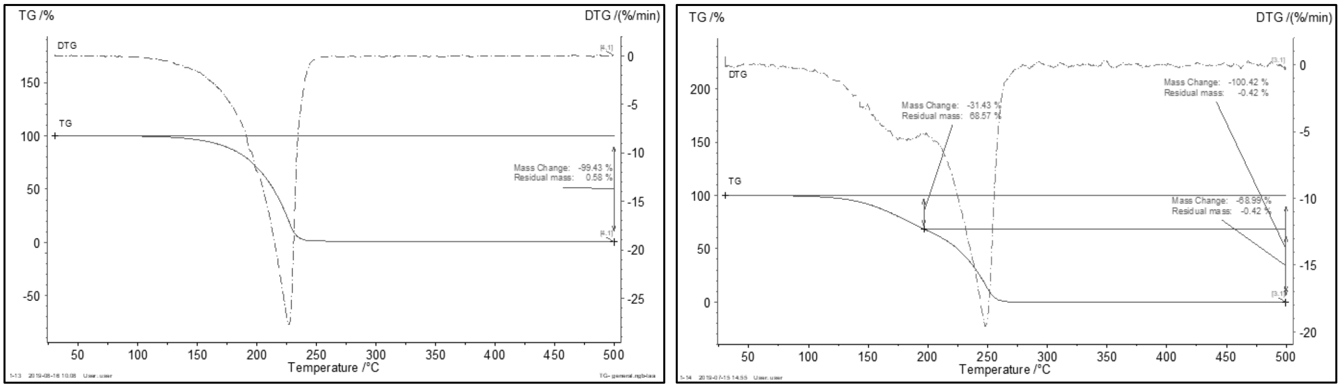

Supplementary Figure 30 TG/DTG curves of $(CNMes)_2$•1,4-FIB (right) and $(CNMes)_2$•1,3,5-FIB (left) adducts

Supplementary References

(1) Aakeroy, C. B., Hurley, E. P. & Desper, J. Modulating Supramolecular Reactivity Using Covalent "Switches" on a Pyrazole Platform. *Cryst. Growth Des.* **12**, 5806-5814, (2012).

(2) Arduengo, A. J., Kline, M., Calabrese, J. C. & Davidson, F. Synthesis of a Reverse Ylide from a Nucleophilic Carbene. *J. Am. Chem. Soc.* **113**, 9704-9705, (1991).

(3) Hopkinson, M. N., Richter, C., Schedler, M. & Glorius, F. An overview of N-heterocyclic carbenes. *Nature* **510**, 485-496, (2014).

(4) Desiraju, G. R. et al. Definition of the halogen bond (IUPAC Recommendations 2013). *Pure Appl. Chem.* **85**, 1711-1713, (2013).

(5) Bader, R. F. W. A Quantum-Theory Of Molecular-Structure And Its Applications. *Chem. Rev.* **91**, 893-928, (1991).

(6) Zhao, Y. & Truhlar, D. G. The M06 suite of density functionals for main group thermochemistry, thermochemical kinetics, noncovalent interactions, excited states, and transition elements: two new functionals and systematic testing of four M06-class functionals and 12 other functionals. *Theor. Chem. Acc.* **120**, 215-241, (2008).

(7) Stevens, W. J., Krauss, M., Basch, H. & Jasien, P. G. Relativistic Compact Effective Potentials And Efficient, Shared-Exponent Basis-Sets For The 3rd-Row, 4th-Row, And 5th-Row Atoms. *Can. J. Chem.* **70**, 612-630, (1992).

(8) Stevens, W. J., Basch, H. & Krauss, M. Compact Effective Potentials And Efficient Shared-Exponent Basis-Sets For The 1st-Row And 2nd-Row Atoms. *J. Chem. Phys.* **81**, 6026-6033, (1984).

(9) Frisch, M. J., Trucks, G. W., Schlegel, H. B., Scuseria, G. E., Robb, M. A., Cheeseman, J. R., Scalmani, G., Barone, V., Mennucci, B., Petersson, G. A., Nakatsuji, H., Caricato, M., Li, X., Hratchian, H., P., Izmaylov, A. F., Bloino, J., Zheng, G., Sonnenberg, J. L., Hada, M., Ehara, M., Toyota, K., Fukuda, R., Hasegawa, J., Ishida, M., Nakajima, T., Honda, Y., Kitao, O., Nakai, H., Vreven, T., Montgomery, J. A., Peralta, J. E., Ogliaro, F., Bearpark, M., Heyd, J. J., Brothers, E., Kudin, N., K., Staroverov, V. N., Kobayashi, R., Normand, J., Raghavachari, K., Rendell, A., Burant, J. C., Iyengar, S. S., Tomasi, J., Cossi, M., Rega, N., Millam, J. M., Klene, M., Knox, J. E., Cross, J. B., Bakken, V., Adamo, C., Jaramillo, J., Gomperts, R., Stratmann, R. E., Yazyev, O., Austin, A. J., Cammi, R., Pomelli, C., Ochterski, J. W., Martin, R. L., Morokuma, K., Zakrzewski, V. G., Voth, G. A., Salvador, P., Dannenberg, J. J., Dapprich, S., Daniels, A. D., Farkas, J., Foresman, B., Ortiz, J. V., Cioslowski, J., Fox, D. J., Gaussian 09, Revision C.01; Gaussian, Inc.: Wallingford CT, (2010).

(10) Espinosa, E., Molins, E. & Lecomte, C. Hydrogen bond strengths revealed by topological analyses of experimentally observed electron densities. *Chem. Phys. Lett.* **285**, 170-173, (1998).

(11) Vener, M. V., Egorova, A. N., Churakov, A. V. & Tsirelson, V. G. Intermolecular hydrogen bond energies in crystals evaluated using electron density properties: DFT computations with periodic boundary conditions. *J. Comput. Chem.* **33**, 2303-2309, (2012).

(12) Bartashevich, E. V. & Tsirelson, V. G. Interplay between non-covalent interactions in complexes and crystals with halogen bonds. *Russ. Chem. Rev.* **83**, 1181-1203, (2014).

(13) Mayer, I. Bond order and valence indices: A personal account. *J. Comput. Chem.* **28**, 204-221, (2007).

(14) Georgiou, D. C., Butler, P., Browne, E. C., Wilson, D. J. D. & Dutton, J. L. On the Bonding in Bis-pyridine Iodonium Cations. *Aust. J. Chem.* **66**, 1179-1188, (2013).

(15) Glendening, E. D., Landis, C. R. & Weinhold, F. Natural bond orbital methods. *Wiley Interdiscip. Rev.: Comput. Mol. Sci.* **2**, 1-42, (2012).

(16) Kerr, J. A. Bond Dissociation Energies By Kinetic Methods. *Chem. Rev.* **66**, 465-500, (1966).

(17) http://www.chemcraftprog.com

(18) Politzer, P. & Murray, J. S. sigma-holes and -holes: Similarities and differences. *J. Comput. Chem.* **39**, 464-471, (2018).
(19) Uson, R., Laguna, A., Laguna, M., Briggs, D.A., Murray, H.H. and Fackler, J.P., Jr. (Tetrahydrothiophene)Gold(I) or Gold(III) Complexes. *Inorg. Synth.* **26**, (1989).
(20) Kukushkin, V. Yu. et al. Synthesis of halogen-bridged complexes $[Pt(R_2SO)(\mu\text{-}X)X]_2$ by thermolysis of *cis*-$[Pt(R_2SO)(R'CN)X_2]$. X-ray structure of di-μ-chlorodichlorobis-(diethyl sulfoxide)diplatinum(II), *Inorg. Chim. Acta*. **183**, 57–63, (1991).
(21) Svensson, P., Lövqvist, K. Kukushkin, V. Yu. & Oskarsson, Å.Thermal *cis* to *trans* isomerization of $[PtCl_2(EtCN)_2]$ and crystal structures of the *cis*- and *trans*-isomers, *Acta Chem. Scand*. **49**, 72–75, (1995).
(22) Hashmi, A. S. K. et al. Carbenes Made Easy: Formation of Unsymmetrically Substituted N-Heterocyclic Carbene Complexes of Palladium(II), Platinum(II) and Gold(I) from Coordinated Isonitriles and their Catalytic Activity. *Adv. Synth. Catal.* **352**, 3001-3012, (2010).
(23) Kinzhalov, M. A., Zolotarev, A. A. & Boyarskiy, V. P. Crystal Structure of cis- $PdCl_2$(CNMes)(2). *J. Struct. Chem.* **57**, 822-825, (2016).
(24) Kinzhalov, M. A., Kashina, M. V., Mikherdov, A. S., Katkova, S. A. & Suslonov, V. V. Synthesis of Platinum(II) Phoshine Isocyanide Complexes and Study of Their Stability in Isomerization and Ligand Disproportionation Reactions. *Russ. J. Gen. Chem.* **88**, 1180-1187, (2018).
(25) Garcia-Gonzalez, M. C., Aguilar-Granda, A., Zamudio-Medina, A., Miranda, L. D. & Rodriguez-Molina, B. Synthesis of Structurally Diverse Emissive Molecular Rotors with Four-Component Ugi Stators. *J. Org. Chem.* **83**, 2570-2581, (2018).
(26) Koppe, K., Haner, J., Mercier, H. P. A., Frohn, H. J. & Schrobilgen, G. J. Xenon(IV)-Carbon Bond of C6F5XeF2 (+); Structural Characterization and Bonding of $[C_6F_5XeF_2]BF_4$, $C_6F_5XeF_2\}BF_4$•2HF, and $C_6F_5XeF_2$ BF_4•nNCCH(3) (n=1, 2); and the Fluorinating Properties of C6F5XeF2 BF4. *Inorg. Chem.* **53**, 11640-11661, (2014).
(27) De Santis, A. et al. N•••Br halogen bonding: One-dimensional infinite chains through the self-assembly of dibromotetrafluorobenzenes with dipyridyl derivatives. *Chem. – Eur. J.* **9**, 3974-3983, (2003).
(28) Yan, D. P. et al. A Cocrystal Strategy to Tune the Luminescent Properties of Stilbene-Type Organic Solid-State Materials. *Angew. Chem. Int. Ed.* **50**, 12483-12486, (2011).
(29) Lisac, K. et al. Halogen-bonded cocrystallization with phosphorus, arsenic and antimony acceptors. *Nat. Commun.* **10**, 61 (2019).
