# Supplementary material for: The halogen bond with isocyano carbon reduces isocyanide odor
Source: Nat Commun. 2020 Jun 10;11:2921. doi: 10.1038/s41467-020-16748-x (PMC7286913; doi:10.1038/s41467-020-16748-x)
Supplement: Supplementary file 3 — Description of Additional Supplementary Files [file 41467_2020_16748_MOESM3_ESM.pdf]

Description of Additional Supplementary Files

File Name: Supplementary Data 1
Description: the geometry of optimized gas phase equilibrium structure of (IAd)•IPFB adduct as XYZ file

File Name: Supplementary Data 2
Description: the geometry of optimized gas phase equilibrium structure of (CNMes)•IPFB adduct as XYZ file
